# Exposure to School Racial Segregation and Late-Life Cognitive Outcomes

**DOI:** 10.1001/jamanetworkopen.2024.52713

**Published:** 2025-01-03

**Authors:** Zhuoer Lin, Yi Wang, Thomas M. Gill, Xi Chen

**Affiliations:** 1 Division of Health Policy and Administration, School of Public Health, University of Illinois Chicago, Chicago; 2 Department of Health Policy and Management, Yale School of Public Health, New Haven, Connecticut; 3 Department of Internal Medicine, Yale School of Medicine, New Haven, Connecticut; 4 Department of Economics, Yale University, New Haven, Connecticut; 5 Yale Alzheimer’s Disease Research Center, New Haven, Connecticut

## Abstract

**Importance:**

Disparities in cognition, including dementia occurrence, persist between non-Hispanic Black (hereinafter, Black) and non-Hispanic White (hereinafter, White) older adults, and are possibly influenced by early educational differences stemming from structural racism. However, the association between school racial segregation and later-life cognition remains underexplored.

**Objective:**

To investigate the association between childhood contextual exposure to school racial segregation and cognitive outcomes in later life.

**Design, Setting, and Participants:**

This cross-sectional study examined a nationally representative sample of US older adults from the Health and Retirement Study. Both restricted childhood residence data and publicly available cognitive assessment data (survey years 1995-2018) were used for Black and White participants aged 65 years and older. Data analyses were performed from March 2, 2023, to October 22, 2024.

**Exposures:**

State-level Black and White dissimilarity index for public elementary schools in the late 1960s (range, 0-100) was used to measure school segregation. States were categorized into high segregation (≥83.6) and low segregation (<83.6) based on the top quintile.

**Main Outcomes and Measures:**

Cognitive scores, cognitive impairment, and dementia were assessed using the Telephone Interview for Cognitive Status and proxy assessment. Multilevel regression analyses were conducted stratified by race and ethnicity, adjusting for sociodemographic covariates. Potential early-life and midlife mediators, including educational attainment, were assessed.

**Results:**

The study sample included 3566 Black (16 104 observations) and 17 555 White (90 874 observations) participants. The mean (SD) age of the sample was 75.6 (7.5) years, and 62 187 (58.1%) were female. Participants exposed to high vs low segregation exhibited lower cognitive scores (13.6 vs 14.5) and a higher prevalence of cognitive impairment (37.0% vs 28.0%) and dementia (14.1% vs 9.3%). Multilevel analyses revealed a significant negative association between school segregation and later-life cognitive outcomes among Black participants, but not among White participants, after adjusting for covariates. Potential mediators across the life course, including educational attainment, explained 57.6% to 72.6% of the association, yet the findings were significant among Black participants for all outcomes. In the model including all mediators and covariates, Black participants exposed to high segregation exhibited significantly lower cognitive scores (coefficient, −0.26; 95% CI, −0.43 to −0.09) and a higher likelihood of cognitive impairment (adjusted odds ratio [AOR], 1.35; 95% CI, 1.12-1.63) and dementia (AOR, 1.26; 95% CI, 1.03-1.54).

**Conclusions and Relevance:**

This cross-sectional study of Black and White older individuals found that childhood exposure to school segregation was associated with late-life cognition among the Black population. Given the increasing amount of school segregation in the US, educational policies aimed at reducing segregation are needed to address health inequities. Clinicians may leverage patients’ early-life educational circumstances to promote screening, prevention, and management of cognitive disorders.

## Key Points

**Question:**

Is school racial segregation during childhood associated with cognitive outcomes in later life among non-Hispanic Black and non-Hispanic White US residents?

**Findings:**

In this cross-sectional study of a nationally representative sample of 21 121 persons, Black individuals exposed to high levels of school segregation had lower cognitive scores and a higher likelihood of cognitive impairment and dementia in later life than those with low exposure. These findings were significant after accounting for a comprehensive array of covariates and life-course mediators; no associations were found among White individuals.

**Meaning:**

These findings suggest that strengthened efforts to reduce school racial segregation could have lasting benefits for cognitive health and advance racial equity, particularly given the enduring segregation of schools as a prominent form of structural racism in the US.

## Teaser

This cross-sectional study examines whether experiencing high levels of racial segregation in school is associated with cognition and dementia among older Black and White adults.

## Introduction

Cognitive impairment poses considerable challenges for older adults,^1^ with Alzheimer disease and related dementias affecting millions of US residents and the burden escalating as the population ages. Marked racial and ethnic disparities persist.^2^ Cognitive disorders disproportionally impact disadvantaged populations, diminishing individual well-being and imposing substantial burdens on caregivers and families, thereby exacerbating societal racial and ethnic disparities.^3^

Emerging evidence underscores the profound influence of adverse early-life circumstances on brain development and cognitive decline over the lifespan.^4,5,6^ Racial differences in early educational environments, particularly those rooted in structural racism, appear to be pivotal in shaping cognition in later life.^7,8,9^

School racial segregation (hereinafter, school segregation), a major aspect of US education systems, may exert particularly profound effects on cognition.^10^ This practice physically segregates students in educational institutions based on racial backgrounds, resulting in vastly unequal educational experiences, qualities, and opportunities between White and minoritized populations. Despite the historic *Brown v Board of Education* ruling, US schools continue to struggle with heightened levels of segregation,^11,12^ with more than half of students attending schools in districts that have predominantly White or racial and ethnic minority group populations, and approximately 40% of Black students attending schools with populations that are 90% to 100% racial and ethnic minority groups.^13,14^

Understanding the long-term association between school segregation and later-life cognition is crucial, as the school environment not only influences educational outcomes but also shapes the quality of educational experience.^15^ Individuals exposed to school segregation typically experience higher rates of discriminatory discipline that may contribute to increased stress levels. Their limited access to educational resources can adversely affect learning opportunities and activities. These disadvantaged school environments may hinder neurologic development through mechanisms such as chronic stress or metabolic dysregulation and processes that impair cognitive ability into later life.^16,17^ However, school segregation could also impact the health of Black children by reducing their exposure to interpersonal racism from White peers, staff, parents, or teachers, especially in predominantly White schools.^18,19,20^ Overall, segregated schools often entail more exposure to discrimination, racism, reduced school resources, and other adversities for Black children, which exacerbate their gaps in cognitive outcomes with White children.^21,22^

Studies evaluating the association between US school segregation and health outcomes in later life have been limited by a singular focus on indirect measures of segregation,^23^ reliance on self-reported data,^24,25,26^ a lack of nationally representative samples,^24,27,28^ and inattention to later-life cognitive outcomes.^18,29,30^ To bridge these gaps, we examined whether childhood contextual exposure to school segregation is associated with cognitive outcomes in later life and explored the potential mediating role of early-life and midlife modifiable risk factors for dementia. Linking historical data on Black and White school segregation in public elementary schools from the late 1960s and early 1970s to a nationally representative sample of US older adults in the Health and Retirement Study (HRS), we hypothesized that childhood exposure to high levels of school segregation is associated with poorer later-life cognitive outcomes, especially among Black individuals. We also hypothesized that the associations between school segregation and later-life cognitive outcomes can be partially mediated by important modifiable factors, such as educational attainment.

## Methods

This study was waived from institutional board review and the requirement for informed consent by the Human Research Protection Program at Yale University, because it was considered not to involve human participation due to its use of secondary data. The study adhered to the Strengthening the Reporting of Observational Studies in Epidemiology (STROBE) reporting guideline.

### Data and Study Participants

Data were derived from 2 primary sources: school segregation data from the Office for Civil Rights (OCR) and longitudinal survey data from the HRS.^31,32^ The school segregation data were obtained from OCR files that included school enrollment statistics and segregation index for US school districts across non-Hispanic Black (hereinafter, Black) and non-Hispanic White (hereinafter, White) populations. Previous studies have thoroughly cleaned and validated the OCR data, constructing segregation index measures at the metropolitan level from the late 1960s.^31,32^ These metropolitan-level public elementary school enrollment and segregation index data spanning 328 US metropolitan areas were used to construct a segregation index for each state.^32^

The HRS is a nationally representative longitudinal survey of US residents aged 50 years or older, with a consistent collection of data on cognition and individual-level sociodemographic and health characteristics since 1995. For this study, we focused on Black and White participants aged 65 years or older surveyed during calendar years 1995-2018 (ie, the most recent wave before the COVID-19 pandemic^33^).

**Figure 1.**
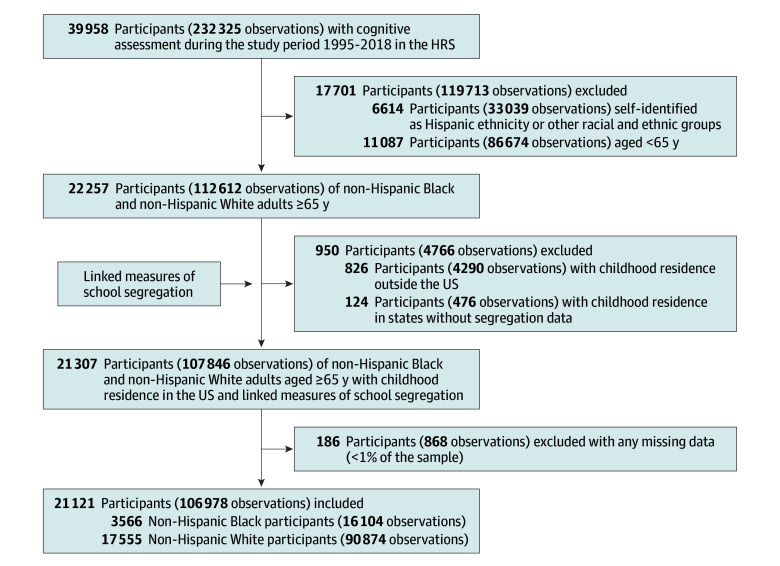
Flowchart of Sample Selection Process HRS indicates Health and Retirement Study.

The sample selection process is shown in Figure 1. Over the study period (1995-2018), 39 958 HRS participants underwent cognitive assessments. We excluded 6614 participants self-identified as Hispanic or racial and ethnic groups other than Black or White and 11 087 participants younger than 65 years, resulting in 22 257 Black and White participants aged 65 years or older. Among them, 21 307 participants with childhood residence in the US and linked measures of school segregation were included. After excluding 186 participants with any missing data (<1% of the sample), the final sample comprised 21 121 (3566 Black and 17 555 White) participants aged 65 years or older with complete data and measurements, contributing to a total of 106 978 observations (16 104 Black and 90 874 White observations).

### Cognitive Outcomes

Cognition was assessed using the Telephone Interview for Cognitive Status, a 27-point cognitive scale encompassing a series of cognitive questions evaluating memory, working memory, and speed of mental processing. The scale reflects global cognitive function, with higher scores indicating better cognitive performance. Cognitive impairment and dementia status were determined based on established criteria: a score below 12 indicated cognitive impairment with or without dementia and a score below 7 indicated dementia.^34,35^

For participants unable to complete the cognitive assessment by themselves, an 11-point proxy cognitive scale was constructed to determine cognitive status, with a higher score indicating poorer cognitive function. Cognitive impairment with or without dementia was determined if the proxy score was higher than 2, and dementia status was identified if the proxy score was higher than 5.^34,35^

### School Segregation

School segregation was assessed using the Black and White dissimilarity index (hereafter, dissimilarity index), which measures the extent of segregation between Black and White students in public elementary schools. Scores on this index, ranging from 0 to 100, indicate the percentage of Black children who would need to move to a different school to achieve an equal distribution of Black and White students across schools in a metropolitan area. Higher scores indicate more segregation. The dissimilarity index primarily relied on enrollment data from public elementary schools reported by the OCR in 1968, supplemented by data from 1969 to 1971.^31,32^

Since all study participants attended elementary schools before 1970, a period during which segregation levels saw minimal changes, using late 1960s data to calculate the dissimilarity index is highly relevant.^32,36^ Participants were asked to report the state where they lived at age 10 years. The mean dissimilarity index score was then calculated for each state in the late 1960s, weighted by the enrollment numbers of Black and White students in public elementary schools within the metropolitan areas.

Due to the skewed distribution of index scores (eFigure 1 in Supplement 1), states were categorized based on quintile of scores, a commonly used cutoff in prior contextual-level research in older US residents.^37^ States in the highest quintile (dissimilarity index ≥83.6) were classified as high segregation, while the others were classified as low segregation.

### Covariates and Mediators

Age, sex, parental educational level, and childhood residence in US southern states were included as key sociodemographic covariates.^38,39^ Additionally, we incorporated regional indicators for childhood residence, a birth-year trend indicator, and region-specific birth-year trend interactions to account for potential unobserved geographic and temporal confounders.

Building on earlier research, we also included a series of early-life and midlife mediators that are potentially shaped by childhood experiences and may impact cognition across the life course. Drawing on the *Lancet* Commission on dementia,^6^ we selected educational attainment as the primary early-life mediator. For midlife, we incorporated several leading modifiable risk factors for dementia and exhibited minimal missing data in the HRS, including hypertension, diabetes, heart diseases, psychiatric conditions, obesity, and smoking.^6,40^ The selection is further supported by emerging evidence linking school segregation with educational attainment and health factors. Examining these mediators may therefore imply pivotal mechanisms through which school segregation influences cognition later in life.^5,27,41,42^

### Statistical Analysis

Descriptive statistics were estimated for the entire sample, as well as for subgroups with high vs low levels of segregation. Differences across subgroups were assessed using χ^2^ tests for categorical variables and Welch *t* tests for continuous variables.

To evaluate the association between school segregation and cognitive outcomes in later life, multilevel models were used, with individuals at level 2 and childhood states of residence at level 3. Random intercepts were included at the state level to account for unobserved heterogeneity and differences between states, while individual-level random intercepts addressed within-individual correlations across multiple observations.^43^ Robust SEs, clustered at the state level, were estimated.^44^ Multilevel linear models were used for the continuous outcome (ie, cognitive score) and multilevel logistic models were used for the dichotomous outcomes (ie, cognitive impairment and dementia). Models were estimated separately for Black and White participants.

In the base model (model A), we examined school segregation and cognitive outcomes, adjusting for covariates (ie, age, sex, parental educational level, childhood residence in US southern states, regional indicators for childhood residence, a birth-year trend indicator, and region-specific birth-year trend interactions). In subsequent models, we sequentially included early-life and midlife mediators. Model B included an early-life mediator (ie, educational attainment), while model C additionally included midlife mediators (ie, health factors involving hypertension, diabetes, heart diseases, psychiatric conditions, obesity, and smoking behaviors). Mediation was evaluated using the difference method (percentage reduction), which compares the coefficients from the mediated model (ie, model C) with the unmediated model (ie, model A). The percentage reduction of the coefficients reflects the extent to which mediators explain the association between school segregation and cognitive outcomes.^45,46^

A series of sensitivity analyses was conducted. First, we redefined high level of school segregation using a less extreme cutoff (top tertile instead of top quintile). Second, we used a continuous measure of school segregation (ie, dissimilarity index) instead of a dichotomous classification (ie, low vs high). Third, we restricted the sample to individuals who lived in urban areas more directly exposed to segregation during childhood. Fourth, following the literature, we used a more time-varying, self-reported measure of school segregation from the HRS life history survey (ie, attending segregated schools during primary education) to reexamine the association. Fifth, state-level birth-year trend indicators were added to further account for time-varying confounding at the state level. The eAppendix in Supplement 1 presents details of these sensitivity analyses.

To address biases from sample attrition, inverse probability-of-attrition weights were computed and applied in all models. Data analyses were performed from March 2, 2023, to October 22, 2024. All analyses were performed using Stata, version 17.0 (StataCorp LLC). Statistical tests were 2-sided, with the significance level set at 5%.

## Results

### Sample Characteristics

The study sample included 21 121 participants (106 978 observations), with 3566 Black (16 104 observations) and 17 555 White (90 874 observations) participants (Figure 1). The mean (SD) age of the sample was 75.6 (7.5) years, 62 187 (58.1%) were female, and 44 791 (41.9%) were male. The Table presents descriptive statistics for the overall sample and for subgroups stratified by high vs low level of school segregation. The 2 segregation groups were similar in age but differed in other characteristics. Participants from high-segregation states had a higher proportion of Black participants (26.4% vs 11.2%), lower levels of educational attainment, and a greater prevalence of health conditions, such as hypertension and diabetes, compared with those from low-segregation states (eTable 1 in Supplement 1).

**Table.** Characteristics of Study Sample With Low and High Levels of School Segregation in the HRS (1995-2018)^a^

Characteristic	Observations, No. (%)		
	Overall (N = 106 978)	Segregation	
		Low (n = 80 127)	High (n = 26 851)
School segregation			
Dissimilarity index, mean (SD)^b^	79.9 (5.7)	77.5 (4.6)	86.9 (1.9)
Cognitive outcomes			
Cognitive score, mean (SD)^c^	14.3 (4.6)	14.5 (4.5)	13.6 (4.9)
Cognitive impairment	32 356 (30.2)	22 432 (28.0)	9924 (37.0)
Dementia	11 238 (10.5)	7448 (9.3)	3790 (14.1)
Covariates			
Age, mean (SD), y	75.6 (7.5)	75.7 (7.5)	75.6 (7.7)
Sex			
Female	62 187 (58.1)	46 341 (57.8)	15 846 (59.0)
Male	44 791 (41.9)	33 786 (42.2)	11 005 (41.0)
Race			
Non-Hispanic Black	16 104 (15.1)	9003 (11.2)	7101 (26.4)
Non-Hispanic White	90 874 (84.9)	71 124 (88.8)	19 750 (73.6)
Mother’s educational level, y			
<8	23 538 (22.0)	16 358 (20.4)	7180 (26.7)
8-12	62 909 (58.8)	48 177 (60.1)	14 732 (54.9)
>12	9640 (9.0)	7635 (9.5)	2005 (7.5)
Unknown	10 891 (10.2)	7957 (9.9)	2934 (10.9)
Father’s educational level, y			
<8	28 840 (27.0)	20 373 (25.4)	8467 (31.5)
8-12	53 643 (50.1)	41 313 (51.6)	12 330 (45.9)
>12	9549 (8.9)	7667 (9.6)	1882 (7.0)
Unknown	14 946 (14.0)	10 774 (13.4)	4172 (15.5)
Childhood residence in US southern states	38 340 (35.8)	22 446 (28.0)	15 894 (59.2)
Early-life mediators (educational level)			
Years of educational attainment, mean (SD)	12.4 (3.0)	12.6 (2.8)	11.8 (3.4)
Midlife mediators (health factors)			
Hypertension	65 811 (61.5)	48 732 (60.8)	17 079 (63.6)
Diabetes	22 332 (20.9)	16 469 (20.6)	5863 (21.8)
Heart diseases	34 440 (32.2)	25 701 (32.1)	8739 (32.5)
Psychiatric conditions	15 447 (14.4)	11 450 (14.3)	3997 (14.9)
Obesity	36 569 (34.2)	26 917 (33.6)	9652 (35.9)
Smoking			
Never smoking	45 996 (43.0)	33 616 (42.0)	12 380 (46.1)
Ever smoking	51 214 (47.9)	39 341 (49.1)	11 873 (44.2)
Currently smoking	9768 (9.1)	7170 (8.9)	2598 (9.7)

Abbreviation: HRS, Health and Retirement Study.

^a^ Differences in characteristics between samples with high and low levels of school segregation were assessed using χ^2^ tests for categorical variables and Welch *t* tests for continuous variables. The test results are presented in eTable 1 in Supplement 1.

^b^ Dissimilarity index possible score, 0 to 100, with higher scores indicating more segregation.

^c^ Cognitive level scale possible score 0 to 27, with higher scores indicating better cognitive performance.

The mean (SD) dissimilarity index score, the measure of school segregation, was 79.9 (5.7) overall, with higher levels in participants from highly segregated states (86.9 [1.9]) compared with those from states with low segregation (77.5 [4.6]). Participants exposed to high segregation during childhood exhibited lower cognitive scores (13.6 [4.9] vs 14.5 [4.5]) and a higher likelihood of cognitive impairment (37.0% vs 28.0%) and dementia (14.1% vs 9.3%), compared with their low segregation counterparts. As shown in eFigure 2 and eFigure 3 in Supplement 1, Black participants who experienced higher segregation consistently showed worse cognitive outcomes across ages compared with those exposed to lower segregation. This difference was most pronounced among participants in the highest quintiles of the dissimilarity index (ie, most segregated), with less noticeable differences in the lower quintiles.

### Association Between School Segregation and Cognitive Outcomes

**Figure 2.**
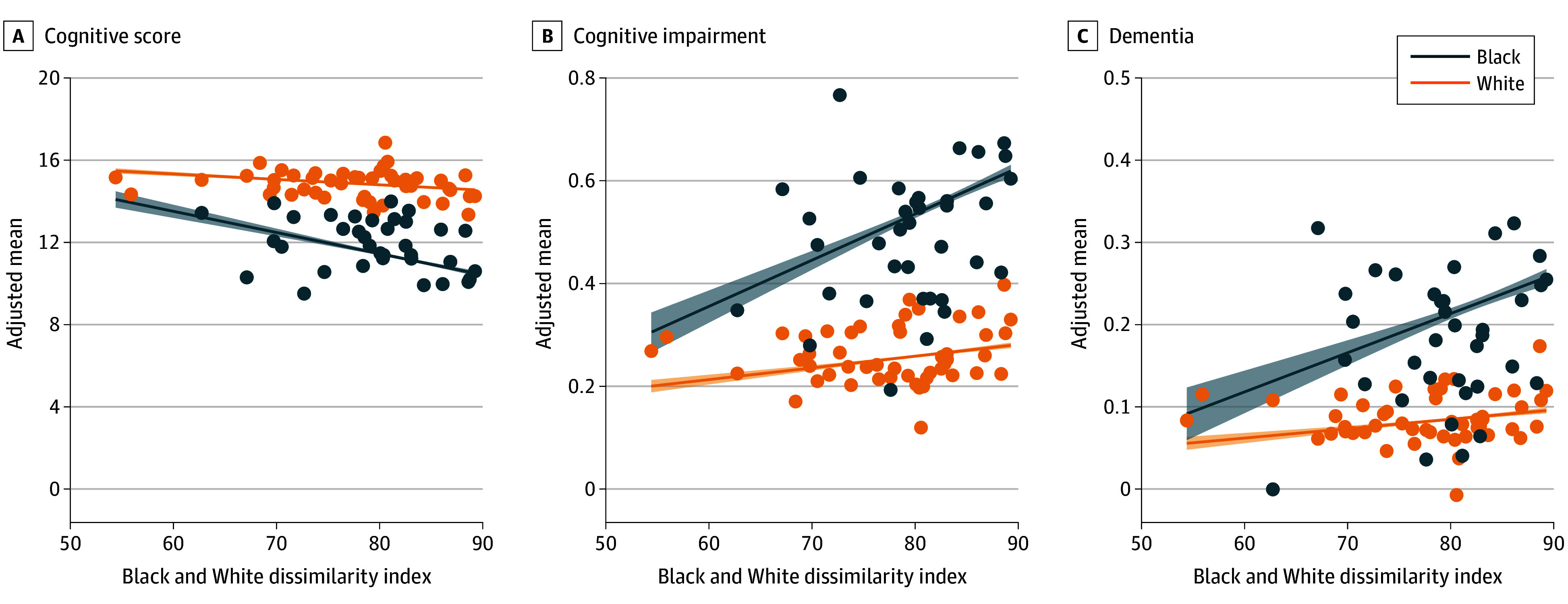
Association Between Black and White Dissimilarity Index and Cognitive Outcomes for Participants in the Health and Retirement Study (HRS) (1995-2018) Inverse associations between school segregation and cognitive outcomes in the HRS are shown. The mean cognitive outcomes were estimated for Black and White participants in each state after adjusting for age and sex; only states with more than 10 observations are plotted. The fitted lines denote the linear association between the Black and White dissimilarity index and adjusted mean cognitive outcomes. Shaded areas indicate 95% CIs.

Figure 2 illustrates the inverse association between state-level dissimilarity index scores and cognition, adjusted for age and sex, separately for Black and White participants. States with higher levels of segregation demonstrated lower mean cognitive scores and higher proportions of cognitive impairment and dementia. There were steeper decreases for Black participants compared with White participants across all cognitive outcomes. Additionally, among Black participants, the decreases were even steeper for those exposed to the highest levels of segregation, indicating disproportionately greater consequences in the most vulnerable individuals (eFigure 4 in Supplement 1).

**Figure 3.**
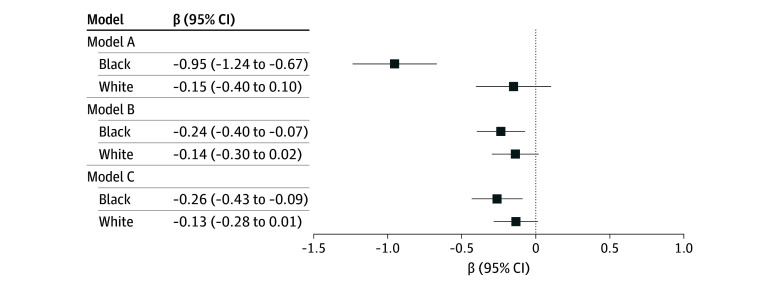
Association Between School Segregation and Cognitive Score for Black and White Participants in the Health and Retirement Study (1995-2018) Estimated Using Multilevel Models Multilevel regression models were used to estimate the association between school segregation and cognitive score for Black and White participants. Descriptions of each model appear in the Methods section. Random intercepts were included at the state level to account for unobserved heterogeneity and differences between states, while individual-level random intercepts addressed within-individual correlations across multiple observations. Robust SEs, clustered at the state level, were estimated accounting for within-state correlation. The detailed numerical estimates and mediation results are presented in eTables 2 and 3 in Supplement 1. Error bars indicate 95% CIs.

**Figure 4.**
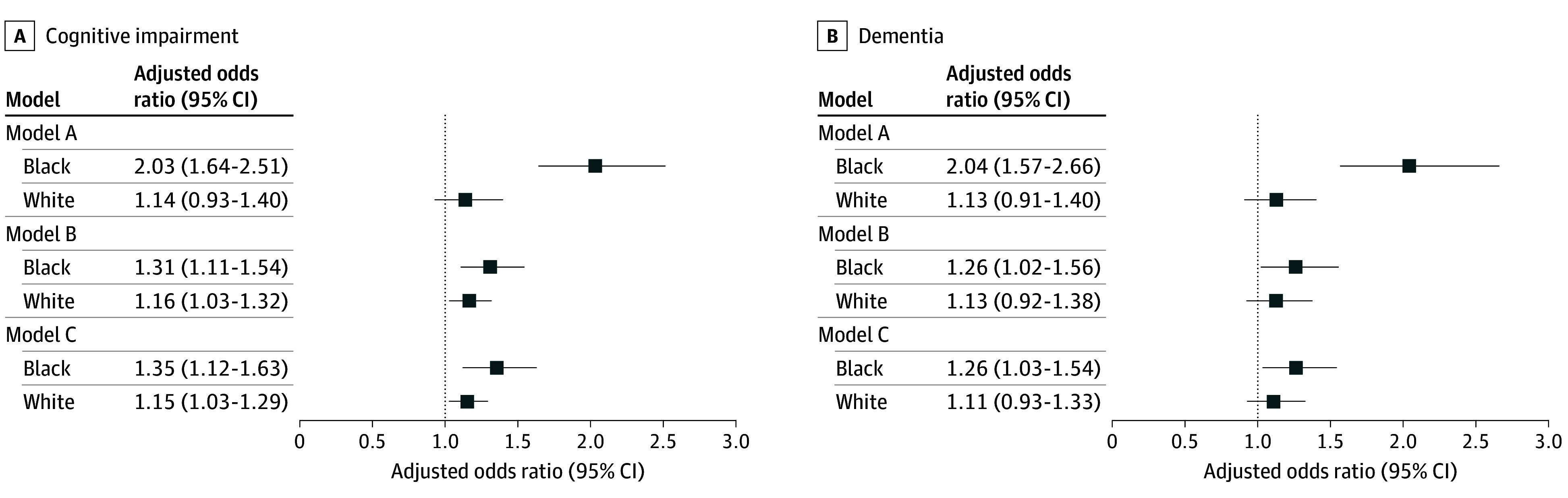
Association Between School Segregation and Cognitive Impairment and Dementia for Black and White Participants in the Health and Retirement Study (1995-2018) Estimated Using Multilevel Models Multilevel regression models were used to estimate the association between school segregation and cognitive impairment and dementia for Black and White participants. Descriptions of each model appear in the Methods section. Random intercepts were included at the state level to account for unobserved heterogeneity and differences between states, while individual-level random intercepts addressed within-individual correlations across multiple observations. Robust SEs, clustered at the state level, were estimated accounting for within-state correlation. The detailed numerical estimates and mediation results are presented in eTables 2 and 3 in Supplement 1.

Multilevel regression analyses, presented in Figure 3 and Figure 4 (with detailed estimates in eTable 2 and eTable 3 in Supplement 1), supported these findings. Overall, participants who experienced high levels of segregation during childhood had lower cognitive scores (Figure 3) and higher odds of cognitive impairment and dementia (Figure 4), compared with those in low-segregation states, after adjusting for covariates (model A). The coefficients and odds ratios were greater and statistically significant for Black participants across all cognitive outcomes, while the associations for White participants were not statistically significant.

The associations observed in model A were partially attenuated after including the early-life mediator, ie, educational attainment (model B), but showed no further attenuation with the addition of midlife health factors (model C). In model C, the associations between school segregation and cognitive outcomes for Black participants remained large and statistically significant. Mediation analysis revealed that early-life and midlife mediators collectively explained 57.6% to 72.6% of the associations. In model C, Black participants exposed to high segregation exhibited significantly lower cognitive scores (coefficient, −0.26; 95% CI, −0.43 to −0.09) and a higher likelihood of cognitive impairment (adjusted odds ratio [AOR], 1.35; 95% CI, 1.12-1.63) and dementia (AOR, 1.26; 95% CI, 1.03-1.54) (Figure 3, Figure 4; eTables 2 and 3 in Supplement 1). Our sensitivity analyses shown in eFigures 5 to 14 in Supplement 1 supported the observed patterns.

## Discussion

Linking a nationally representative sample of US older adults to historical administrative data on segregation, we provide new insights into the long-term association between school segregation exposure and cognitive outcomes in later life. Our findings suggest that childhood exposure to high levels of school segregation was associated with lower cognitive scores and a higher likelihood of cognitive impairment and dementia among Black individuals.

Despite decades of desegregation efforts, school segregation persists,^47,48^ and its long-term health consequences have not been thoroughly investigated due to data constraints. By using historical administrative records of school segregation rather than relying on self-reports, our study captures a less subjective contextual measure of segregation exposure. Linking these segregation measures to HRS data enabled us to evaluate important and clinically relevant cognitive outcomes in later life in a nationally representative sample of older adults.

To our knowledge, this is the first study to use such linked data to examine the association between school segregation and various cognitive outcomes in later life. Our findings align with existing research reporting that adverse educational experiences are negatively associated with later-life cognition.^9^ Previous studies have also suggested that higher educational quality is linked to a lower risk of dementia and improved cognitive outcomes.^49,50^ By using comprehensive national datasets, our analysis provides broader contextual insights into the association between school segregation and cognition, distinguishing it from studies relying on self-reports,^24,25,26^ regional-specific data,^24,27^ or those not focused on cognition.^18,29,30^

High levels of school segregation are often indicative of systemic educational disparities, where schools with a predominantly Black population receive fewer resources, leading to poorer educational quality. States with higher segregation levels typically allocate less funding to schools serving primarily Black students,^51^ resulting in underresourced schools with higher teacher turnover and larger class sizes.^52,53^ This disparity may impact the educational experiences (eg, limited learning opportunities) and physical development (eg, inadequate nutrition and physical activity) of Black students, which may affect cognitive function later in life.^17,18^ Our findings are consistent with previous research reporting that Black individuals are disproportionately affected by these systemic inequities, leading to worse cognitive outcomes.^9,26,30,39^ In contrast, while White participants may also experience disadvantaged environments with fewer social interactions across diverse racial communities, they are less exposed to the systemic racism that disproportionately impacts Black students, which may explain the lack of negative associations between segregation and cognition in this group.

We found that educational attainment mediated a significant portion of the association between school segregation and cognitive outcomes. School segregation has been shown to reduce educational opportunities for Black students, manifested in lower educational attainment.^54^ As educational attainment is a key modifiable risk factor for dementia, it likely influences cognitive development in early life and affects cognitive outcomes through various pathways over the life course.^6^ For example, individuals with lower educational levels may have reduced access to health care or may adopt unhealthy behaviors, such as smoking, further increasing their risks of cognitive disorders. Our findings suggest that policies addressing educational inequities, particularly in highly segregated states, could reap long-term benefits for reducing health disparities. Moreover, identifying people at risk of dementia in the clinical settings, including using information on their early-life schooling, might lower the bar for cognitive screening or testing, which could help prioritize limited clinical resources for higher-risk groups.

Although midlife health conditions and behaviors, such as hypertension, diabetes, and smoking, are known to influence cognition in later life,^6^ they did not further attenuate the association between school segregation and cognitive outcomes. This may be because educational attainment already encapsulates much of the impact of these midlife risk factors on cognition. Future research should explore more detailed measures to better understand the mechanisms.

### Limitations

Our study has limitations. First, state-level segregation measures may not fully capture localized segregation. More granular segregation data could provide a clearer understanding of exposure, particularly considering the rural and urban differences. Second, we do not have data on educational quality, such as teacher qualifications, class sizes, and school duration, which could elucidate the mechanisms linking segregation to cognition. Additionally, lack of state-level data for cohorts prior to the 1970s limits our ability to implement more comprehensive regional controls. Third, because evaluating cognitive trajectories was beyond the scope of this study, future research should explore the association between school segregation and cognitive decline. Fourth, although we accounted for proxy respondents when evaluating cognitive impairment and dementia, the evaluation of cognitive scores was limited to self-respondents. Future studies should consider combining self- and proxy-reported scores for more comparable results.^55^ Fifth, the HRS does not include data on early brain structure or function, limiting the exploration of biological pathways linking early-life segregation to cognitive aging. More comprehensive chain mediation analyses could further deepen understanding. Sixth, while our study shows associations between segregation and cognition, as well as the mediating role of life course factors, the observational study design cannot assess causality.

## Conclusions

Our cross-sectional study highlights the long-term neurologic consequences of school segregation. We observed that high levels of school segregation are associated with poorer cognitive outcomes in later life, suggesting that structural racism in education has long-term outcomes associated with cognition. Reducing school segregation and addressing educational inequities could have profound benefits slowing cognitive aging and mitigating racial health disparities. Our findings contribute to the growing evidence on the importance of addressing systemic racism in education to promote health equity and improve health outcomes for historically marginalized populations.

## Notes

Supplement 1. **eFigure 1.** Distribution of School Segregation at the State Level **eFigure 2.** Age Trend in Cognitive Outcomes by Low vs High Levels of School Segregation for Black and White Participants in the HRS **eFigure 3.** Age Trend in Cognitive Outcomes by Quintiles of Segregation Index for Black and White Participants in the HRS **eFigure 4.** Relationship Between Black and White Dissimilarity Index and Cognitive Outcomes for Black and White Participants in the HRS (Nonlinear) **eFigure 5.** Sensitivity Analysis Using the Highest Tertile as Cutoff for Low vs High Level of Segregation: Association Between School Segregation and Cognitive Score by Race **eFigure 6.** Sensitivity Analysis Using the Highest Tertile as Cutoff for Low vs High Level of Segregation: Association Between School Segregation and Cognitive Impairment and Dementia by Race **eFigure 7.** Sensitivity Analysis Using the Continuous Specification of School Segregation (ie, Black and White Dissimilarity Index): Association Between School Segregation and Cognitive Score by Race **eFigure 8.** Sensitivity Analysis Using the Continuous Specification of School Segregation (ie, Black and White Dissimilarity Index): Association Between School Segregation and Cognitive Impairment and Dementia by Race **eFigure 9.** Sensitivity Analysis With Sample Restricted to Participants Who Lived in Urban Areas During Childhood: Association Between School Segregation and Cognitive Score by Race **eFigure 10.** Sensitivity Analysis With Sample Restricted to Participants Who Lived in Urban Areas During Childhood: Association Between School Segregation and Cognitive Impairment and Dementia by Race **eFigure 11.** Sensitivity Analysis Using Self-Reported Time-Varying Measures of School Segregation Exposure: Association Between School Segregation and Cognitive Score by Race **eFigure 12.** Sensitivity Analysis Using Self-Reported Time-Varying Measures of School Segregation Exposure: Association Between School Segregation and Cognitive Impairment and Dementia by Race **eFigure 13.** Sensitivity Analysis Using Self-Reported Time-Varying Measures of School Segregation Exposure With Additional Adjustment for State-Level Geographical and Temporal Variations: Association Between School Segregation and Cognitive Score by Race **eFigure 14.** Sensitivity Analysis Using Self-Reported Time-Varying Measures of School Segregation Exposure With Additional Adjustment for State-Level Geographical and Temporal Variations: Association Between School Segregation and Cognitive Impairment and Dementia by Race **eTable 1.** Differences in Characteristics Between Sample With High vs Low Level of School Segregation Assessed Using Appropriate Statistical Tests **eTable 2**. Association Between School Segregation and Cognitive Outcomes for Black Participants in the HRS (1995-2018) **eTable 3**. Association Between School Segregation and Cognitive Outcomes for White Participants in the HRS (1995-2018) **eAppendix**. Sensitivity Analyses

Supplement 2.
Data Sharing Statement
