# Supplementary material for: Exposure to School Racial Segregation and Late-Life Cognitive Outcomes
Source: JAMA Netw Open. 2025 Jan 3;8(1):e2452713. doi: 10.1001/jamanetworkopen.2024.52713 (PMC11699536; doi:10.1001/jamanetworkopen.2024.52713)
Supplement: Supplement 1. — eFigure 1. Distribution of School Segregation at the State Level eFigure 2. Age Trend in Cognitive Outcomes by Low vs High Levels of School Segregation for Black and White Participants in the HRS eFigure 3. Age Trend in Cognitive Outcomes by Quintiles of Segregation Index for Black and White Participants in the HRS eFigure 4. Relationship Between Black and White Dissimilarity Index and Cognitive Outcomes for Black and White Participants in the HRS (Nonlinear) eFigure 5. Sensitivity Analysis Using the Highest Tertile as Cutoff for Low vs High Level of Segregation: Association Between School Segregation and Cognitive Score by Race eFigure 6. Sensitivity Analysis Using the Highest Tertile as Cutoff for Low vs High Level of Segregation: Association Between School Segregation and Cognitive Impairment and Dementia by Race eFigure 7. Sensitivity Analysis Using the Continuous Specification of School Segregation (ie, Black and White Dissimilarity Index): Association Between School Segregation and Cognitive Score by Race eFigure 8. Sensitivity Analysis Using the Continuous Specification of School Segregation (ie, Black and White Dissimilarity Index): Association Between School Segregation and Cognitive Impairment and Dementia by Race eFigure 9. Sensitivity Analysis With Sample Restricted to Participants Who Lived in Urban Areas During Childhood: Association Between School Segregation and Cognitive Score by Race eFigure 10. Sensitivity Analysis With Sample Restricted to Participants Who Lived in Urban Areas During Childhood: Association Between School Segregation and Cognitive Impairment and Dementia by Race eFigure 11. Sensitivity Analysis Using Self-Reported Time-Varying Measures of School Segregation Exposure: Association Between School Segregation and Cognitive Score by Race eFigure 12. Sensitivity Analysis Using Self-Reported Time-Varying Measures of School Segregation Exposure: Association Between School Segregation and Cognitive Impairment and Dementia by Race eFigure 1 [file jamanetwopen-e2452713-s001.pdf]

Supplementary Online Content

Lin Z, Wang Y, Gill TM, Chen X. Exposure to school racial segregation and late-life cognitive outcomes. *JAMA Netw Open*. 2024;8(1):e2452713. doi:10.1001/jamanetworkopen.2024.52713

eFigure 1. Distribution of School Segregation at the State Level

eFigure 2. Age Trend in Cognitive Outcomes by Low vs High Levels of School Segregation for Black and White Participants in the HRS

eFigure 3. Age Trend in Cognitive Outcomes by Quintiles of Segregation Index for Black and White Participants in the HRS

eFigure 4. Relationship Between Black and White Dissimilarity Index and Cognitive Outcomes for Black and White Participants in the HRS (Nonlinear)

eFigure 5. Sensitivity Analysis Using the Highest Tertile as Cutoff for Low vs High Level of Segregation: Association Between School Segregation and Cognitive Score by Race

eFigure 6. Sensitivity Analysis Using the Highest Tertile as Cutoff for Low vs High Level of Segregation: Association Between School Segregation and Cognitive Impairment and Dementia by Race

eFigure 7. Sensitivity Analysis Using the Continuous Specification of School Segregation (ie, Black and White Dissimilarity Index): Association Between School Segregation and Cognitive Score by Race

eFigure 8. Sensitivity Analysis Using the Continuous Specification of School Segregation (ie, Black and White Dissimilarity Index): Association Between School Segregation and Cognitive Impairment and Dementia by Race

eFigure 9. Sensitivity Analysis With Sample Restricted to Participants Who Lived in Urban Areas During Childhood: Association Between School Segregation and Cognitive Score by Race

eFigure 10. Sensitivity Analysis With Sample Restricted to Participants Who Lived in Urban Areas During Childhood: Association Between School Segregation and Cognitive Impairment and Dementia by Race

eFigure 11. Sensitivity Analysis Using Self-Reported Time-Varying Measures of School Segregation Exposure: Association Between School Segregation and Cognitive Score by Race

eFigure 12. Sensitivity Analysis Using Self-Reported Time-Varying Measures of School Segregation Exposure: Association Between School Segregation and Cognitive Impairment and Dementia by Race

eFigure 13. Sensitivity Analysis Using Self-Reported Time-Varying Measures of School Segregation Exposure With Additional Adjustment for State-Level Geographical and Temporal Variations: Association Between School Segregation and Cognitive Score by Race

eFigure 14. Sensitivity Analysis Using Self-Reported Time-Varying Measures of School Segregation Exposure With Additional Adjustment for State-Level Geographical and Temporal Variations: Association Between School Segregation and Cognitive Impairment and Dementia by Race

eTable 1. Differences in Characteristics Between Sample With High vs Low Level of School Segregation Assessed Using Appropriate Statistical Tests

eTable 2. Association Between School Segregation and Cognitive Outcomes for Black Participants in the HRS (1995-2018)

eTable 3. Association Between School Segregation and Cognitive Outcomes for White Participants in the HRS (1995-2018)

eAppendix. Sensitivity Analyses

This supplementary material has been provided by the authors to give readers additional information about their work.

© 2025 Lin Z et al. *JAMA Network Open.*

eFigure 1. Distribution of school segregation at the state level

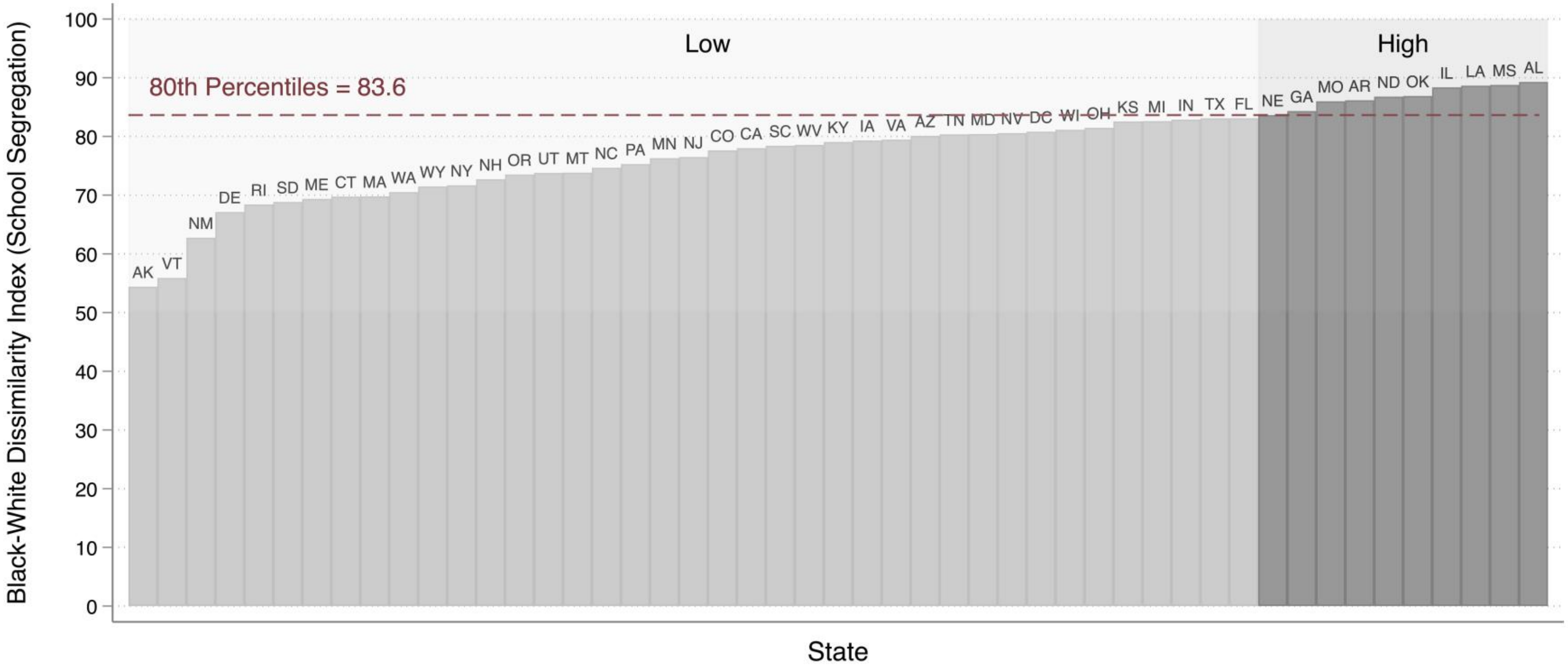

Notes: The figure presents the levels of school segregation in late 1960s measured at the state level. The vertical bar denotes the Black-White dissimilarity index (representing the absolute levels of school segregation) for each state, and states are ordered by their index scores. Standard two-letter state abbreviations are displayed at the top of corresponding bars. States with dissimilarity index higher than 80th percentiles (i.e., dissimilarity index ≥ 83.6) are categorized as "high segregation" (in dark gray color), and otherwise as "low segregation" (in light gray color). Black refers to non-Hispanic Black, and White refers to non-Hispanic White.

© 2025 Lin Z et al. *JAMA Network Open.*

eFigure 2. Age trend in cognitive outcomes by low vs. high levels of school segregation for Black and White participants in the HRS

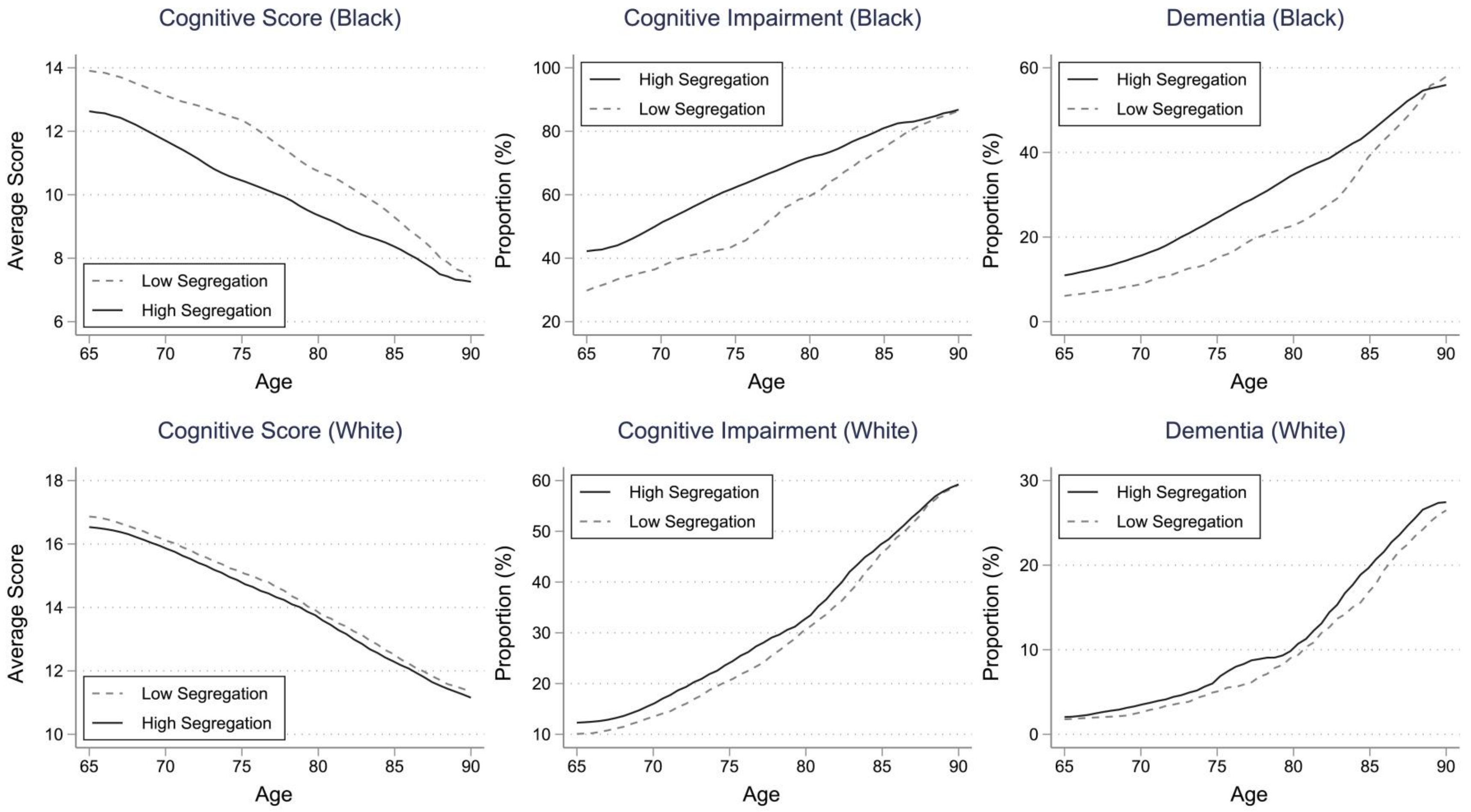

Notes: The figure presents the trajectories of cognitive score, cognitive impairment and dementia across age for Black and White participants in the Health and Retirement Study (HRS, 1995-2018). In each panel, sample were stratified into high vs. low levels of school segregation based on quintiles of Black-White dissimilarity index scores, and the trajectories for each group were fitted and plotted. The lines were fitted using local moving average. Black refers to non-Hispanic Black, White refers to non-Hispanic White.

© 2025 Lin Z et al. *JAMA Network Open.*

eFigure 3. Age trend in cognitive outcomes by quintiles of segregation index for Black and White participants in the HRS

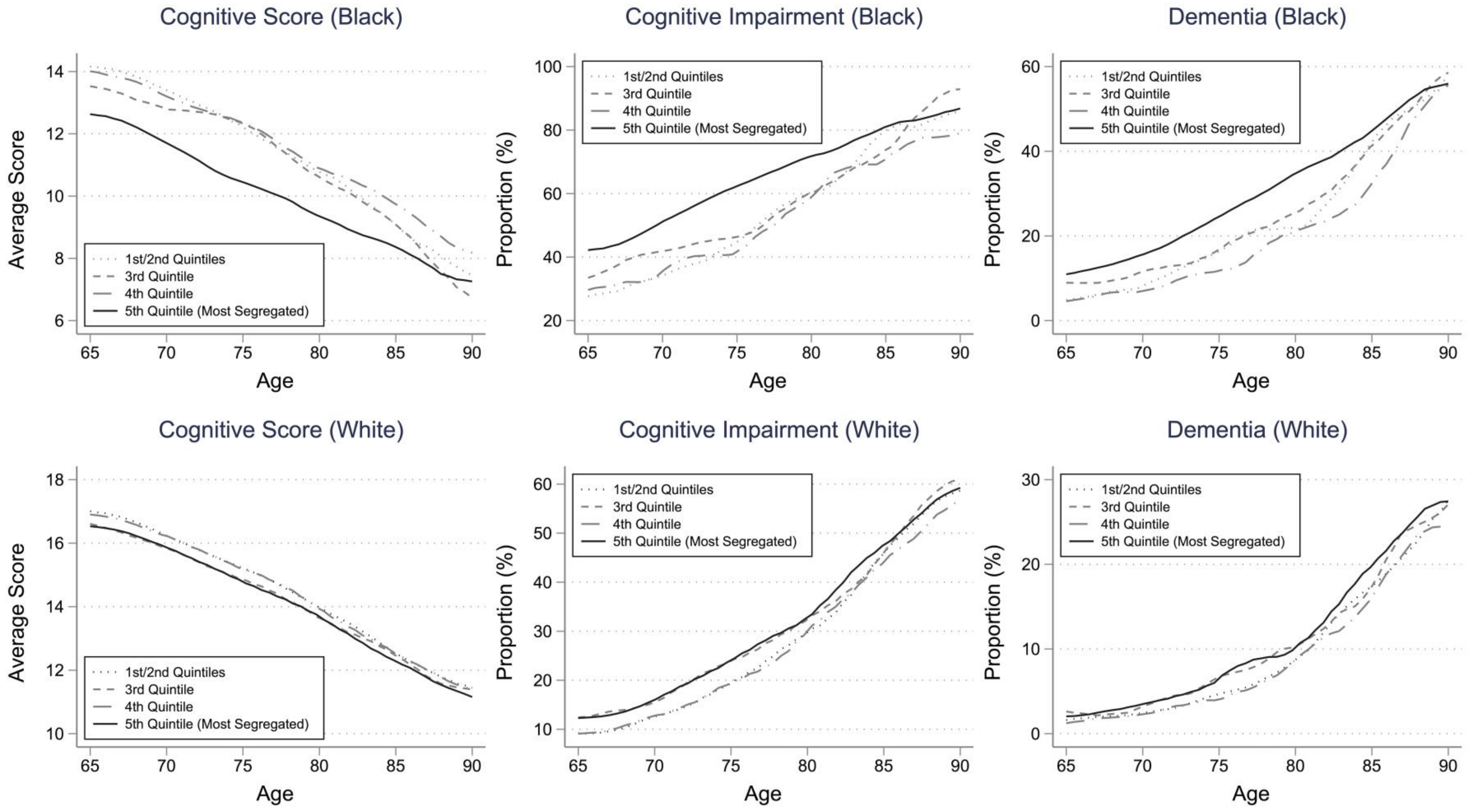

Notes: The figure presents the trajectories of cognitive score, cognitive impairment and dementia across age for Black and White participants in the Health and Retirement Study (HRS, 1995-2018). In each panel, sample were stratified based on quintiles of Black-White dissimilarity index scores, and the trajectories for each group were fitted and plotted. The lines were fitted using local moving average. Black refers to non-Hispanic Black, White refers to non-Hispanic White.

© 2025 Lin Z et al. *JAMA Network Open.*

eFigure 4. Relationship between Black and White dissimilarity index and cognitive outcomes for Black and White participants in the HRS (nonlinear)

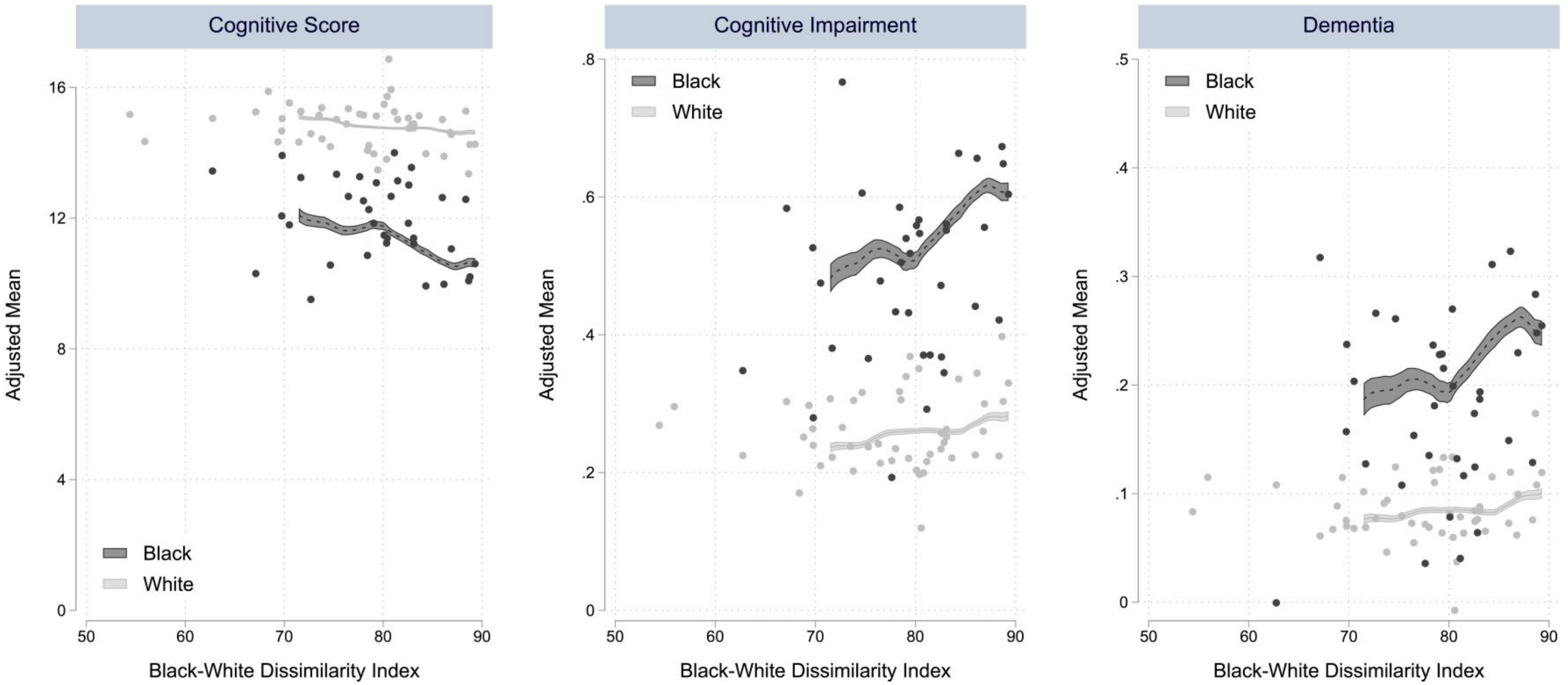

Notes: The figure presents scatterplots of US states demonstrating the inverse relationships between school segregation (measured by Black-White dissimilarity index) and cognitive outcomes in the Health and Retirement Study (HRS, 1995-2018), with dissimilarity index on the x-axis and adjusted cognitive outcomes on the y-axis. The scatterplots are stratified by Black (in black color) and White (in gray color) participants. Black refers to non-Hispanic Black, and White refers to non-Hispanic White. The average cognitive outcomes were estimated respectively for Black and White participants in each state after adjusting for age and sex; and only states with more than 10 observations are plotted. The fitted lines (with 95% CI) denote the non-linear relationship between school segregation and adjusted average cognitive outcomes for Black participants (in black color) and White participants (in gray color), fitted nonparametrically by local moving average.

© 2025 Lin Z et al. *JAMA Network Open.*

eFigure 5. Sensitivity analysis using the highest tertile as cutoff for low vs high level of segregation: association between school segregation and cognitive score by race

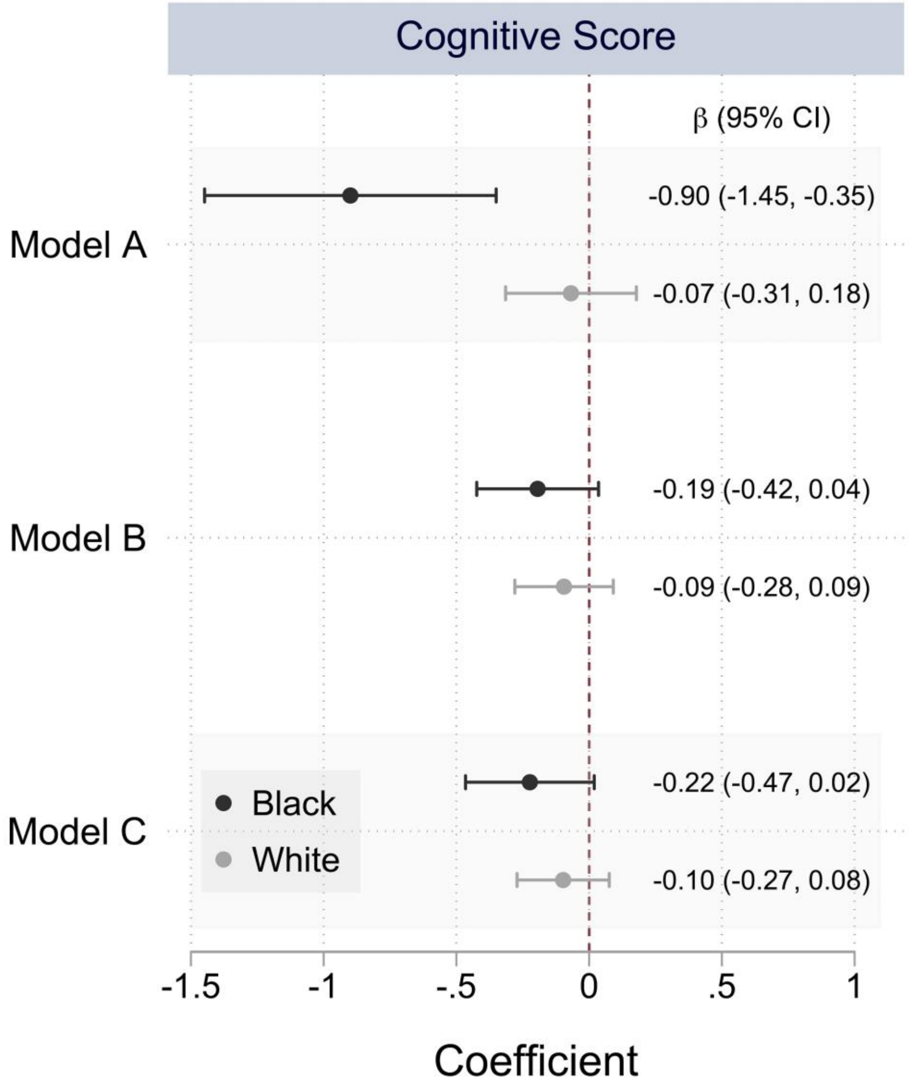

© 2025 Lin Z et al. *JAMA Network Open.*

Notes: Multilevel regression models were used to estimate the association between school segregation and cognitive score for Black (in black color) and White participants (in gray color) in the Health and Retirement Study (HRS, 1995-2018). Black refers to non-Hispanic Black, and White refers to non-Hispanic White. Horizontal lines represent the 95% confidence interval, and numerical estimates are displayed alongside each line. In the sensitivity analyses, we redefined the threshold for high levels of school segregation, using a less extreme cutoff (the top tertile instead of the top quintile). Model A adjusted for covariates, including age, sex, parental education, childhood residence in U.S. Southern States, regional indicators for childhood residence, a birth-year trend indicator, and region-specific birth-year trend interactions. Model B additionally included early-life mediator, i.e., educational attainment. Model C further added mid-life mediators, including health factors involving hypertension, diabetes, heart diseases, psychiatric conditions, obesity, and smoking behaviors. Random intercepts were included at the state level to account for unobserved heterogeneity and differences between states, while individual-level random intercepts addressed within-individual correlations across multiple observations. Robust standard errors, clustered at the state level, were estimated accounting for within-state correlation.

© 2025 Lin Z et al. *JAMA Network Open.*

eFigure 6. Sensitivity analysis using the highest tertile as cutoff for low vs. high level of segregation: association between school segregation and cognitive impairment and dementia by race

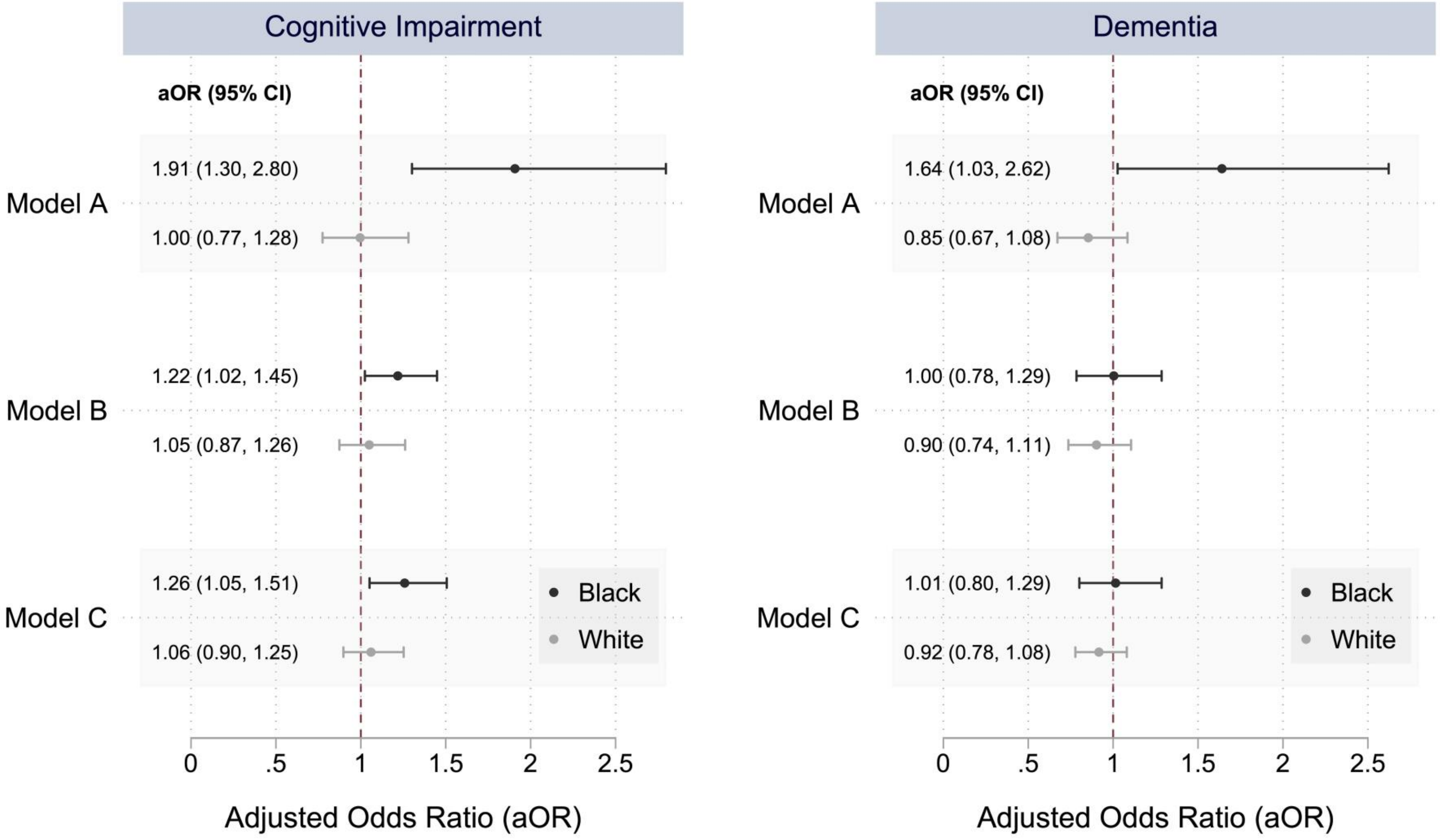

Notes: Multilevel regressions were used to estimate the association between school segregation and cognitive impairment (left panel) and dementia (right panel), for Black (in black color) and White participants (in gray color) in the Health and Retirement Study (HRS, 1995-2018). Black refers to non-Hispanic Black, and White refers to non-Hispanic White. Horizontal lines represent the 95% confidence

© 2025 Lin Z et al. *JAMA Network Open.*

interval, and numerical estimates are displayed alongside each line. In the sensitivity analyses, we redefined the threshold for high levels of school segregation, using a less extreme cutoff (the top tertile instead of the top quintile). Model A adjusted for covariates, including age, sex, parental education, childhood residence in U.S. Southern States, regional indicators for childhood residence, a birth-year trend indicator, and region-specific birth-year trend interactions. Model B additionally included early-life mediator, i.e., educational attainment. Model C further added mid-life mediators, including health factors involving hypertension, diabetes, heart diseases, psychiatric conditions, obesity, and smoking behaviors. Random intercepts were included at the state level to account for unobserved heterogeneity and differences between states, while individual-level random intercepts addressed within-individual correlations across multiple observations. Robust standard errors, clustered at the state level, were estimated accounting for within-state correlation.

© 2025 Lin Z et al. *JAMA Network Open.*

eFigure 7. Sensitivity analysis using the continuous specification of school segregation (i.e., Black-White dissimilarity index): association between school segregation and cognitive score by race

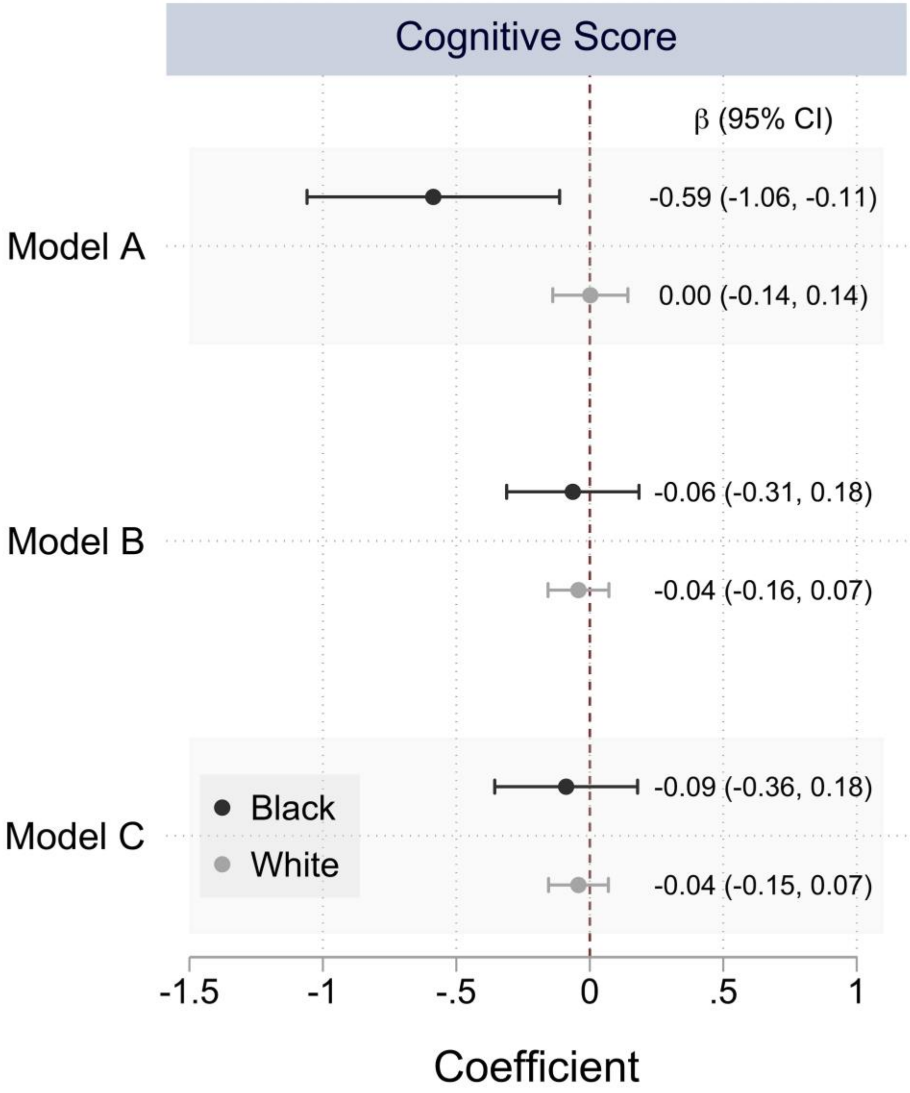

© 2025 Lin Z et al. *JAMA Network Open.*

Notes: Multilevel regression models were used to estimate the association between school segregation and cognitive score for Black (in black color) and White participants (in gray color) in the Health and Retirement Study (HRS, 1995-2018). Black refers to non-Hispanic Black, and White refers to non-Hispanic White. Horizontal lines represent the 95% confidence interval, and numerical estimates are displayed alongside each line. In the sensitivity analyses, we employed a continuous measure of school segregation, represented by the dissimilarity index, rather than using a dichotomous classification (low vs. high segregation). Model A adjusted for covariates, including age, sex, parental education, childhood residence in U.S. Southern States, regional indicators for childhood residence, a birth-year trend indicator, and region-specific birth-year trend interactions. Model B additionally included early-life mediator, i.e., educational attainment. Model C further added mid-life mediators, including health factors involving hypertension, diabetes, heart diseases, psychiatric conditions, obesity, and smoking behaviors. Random intercepts were included at the state level to account for unobserved heterogeneity and differences between states, while individual-level random intercepts addressed within-individual correlations across multiple observations. Robust standard errors, clustered at the state level, were estimated accounting for within-state correlation.

© 2025 Lin Z et al. *JAMA Network Open.*

eFigure 8. Sensitivity analysis using the continuous specification of school segregation (i.e., Black-White dissimilarity index): association between school segregation and cognitive impairment and dementia by race

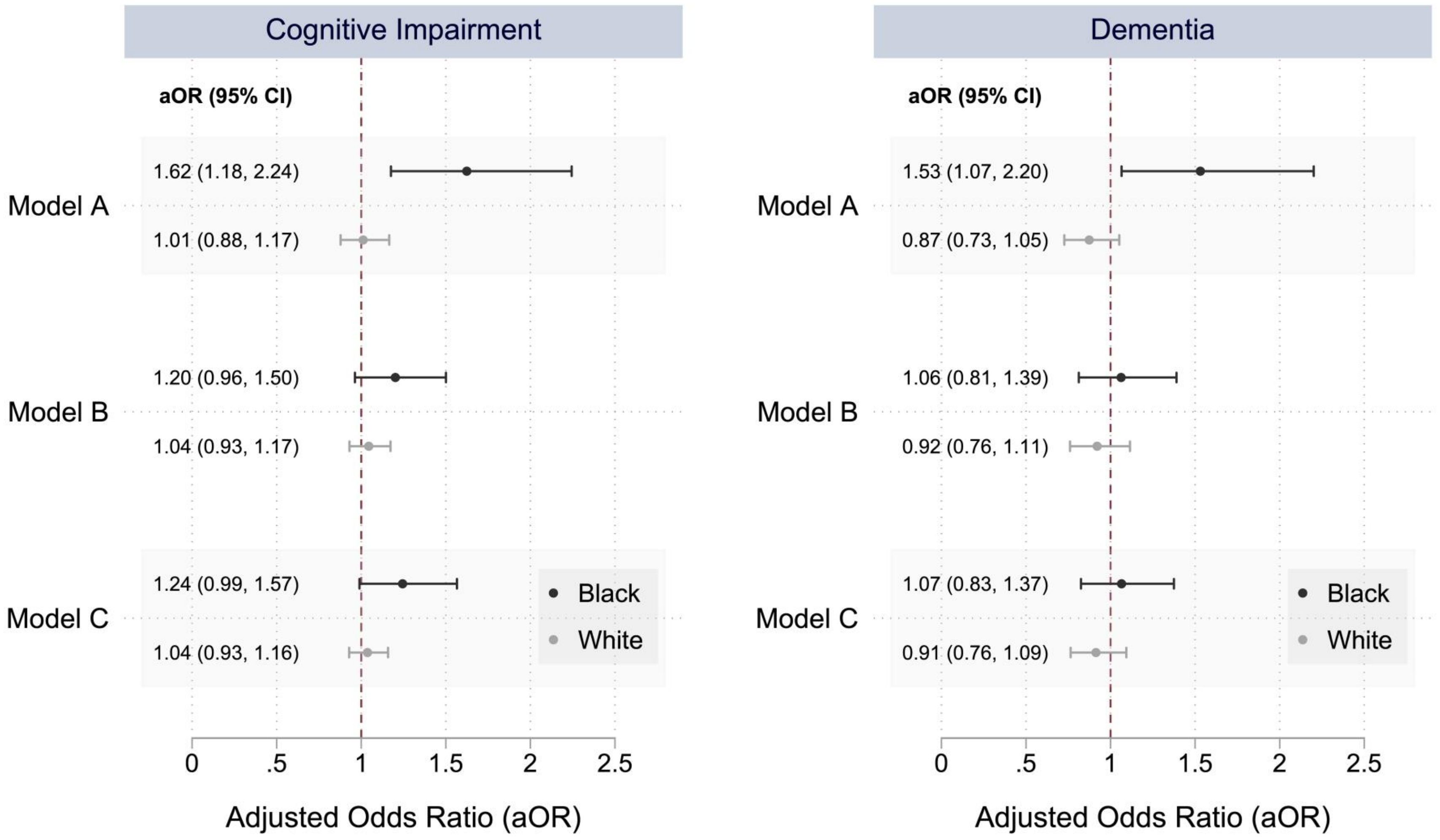

Notes: Multilevel regressions were used to estimate the association between school segregation and cognitive impairment (left panel) and dementia (right panel), for Black (in black color) and White participants (in gray color) in the Health and Retirement Study (HRS, 1995-2018). Black refers to non-Hispanic Black, and White refers to non-Hispanic White. Horizontal lines represent the 95% confidence

© 2025 Lin Z et al. *JAMA Network Open.*

interval, and numerical estimates are displayed alongside each line. In the sensitivity analyses, we employed a continuous measure of school segregation, represented by the dissimilarity index, rather than using a dichotomous classification (low vs. high segregation). Model A adjusted for covariates, including age, sex, parental education, childhood residence in U.S. Southern States, regional indicators for childhood residence, a birth-year trend indicator, and region-specific birth-year trend interactions. Model B additionally included early-life mediator, i.e., educational attainment. Model C further added mid-life mediators, including health factors involving hypertension, diabetes, heart diseases, psychiatric conditions, obesity, and smoking behaviors. Random intercepts were included at the state level to account for unobserved heterogeneity and differences between states, while individual-level random intercepts addressed within-individual correlations across multiple observations. Robust standard errors, clustered at the state level, were estimated accounting for within-state correlation.

© 2025 Lin Z et al. *JAMA Network Open.*

eFigure 9. Sensitivity analysis with sample restricted to participants who lived in urban areas during childhood: association between school segregation and cognitive score by race

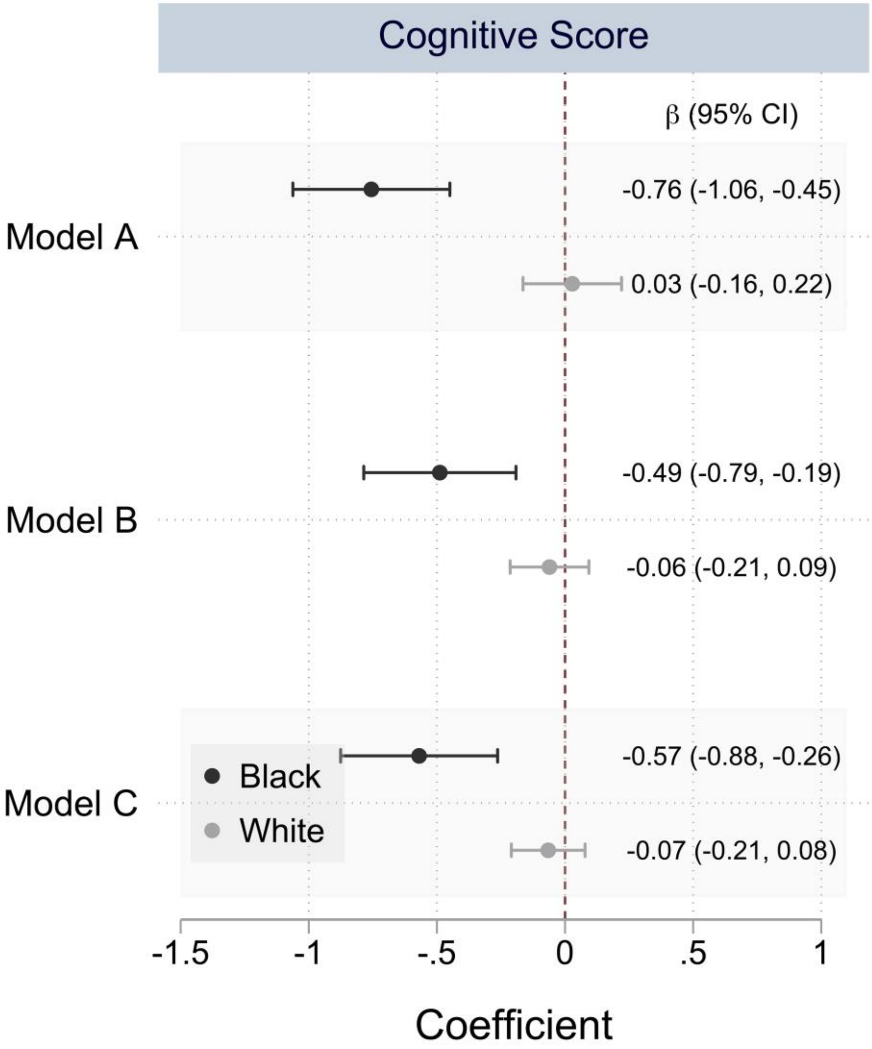

© 2025 Lin Z et al. *JAMA Network Open.*

Notes: Multilevel regression models were used to estimate the association between school segregation and cognitive score for Black (in black color) and White participants (in gray color) in the Health and Retirement Study (HRS, 1995-2018). Black refers to non-Hispanic Black, and White refers to non-Hispanic White. Horizontal lines represent the 95% confidence interval, and numerical estimates are displayed alongside each line. In the sensitivity analyses, we restricted our sample to participants who lived in urban areas during childhood. The segregation measure is the same as the main setting. Model A adjusted for covariates, including age, sex, parental education, childhood residence in U.S. Southern States, regional indicators for childhood residence, a birth-year trend indicator, and region-specific birth-year trend interactions. Model B additionally included early-life mediator, i.e., educational attainment. Model C further added mid-life mediators, including health factors involving hypertension, diabetes, heart diseases, psychiatric conditions, obesity, and smoking behaviors. Random intercepts were included at the state level to account for unobserved heterogeneity and differences between states, while individual-level random intercepts addressed within-individual correlations across multiple observations. Robust standard errors, clustered at the state level, were estimated accounting for within-state correlation.

© 2025 Lin Z et al. *JAMA Network Open.*

eFigure 10. Sensitivity analysis with sample restricted to participants who lived in urban areas during childhood: association between school segregation and cognitive impairment and dementia by race

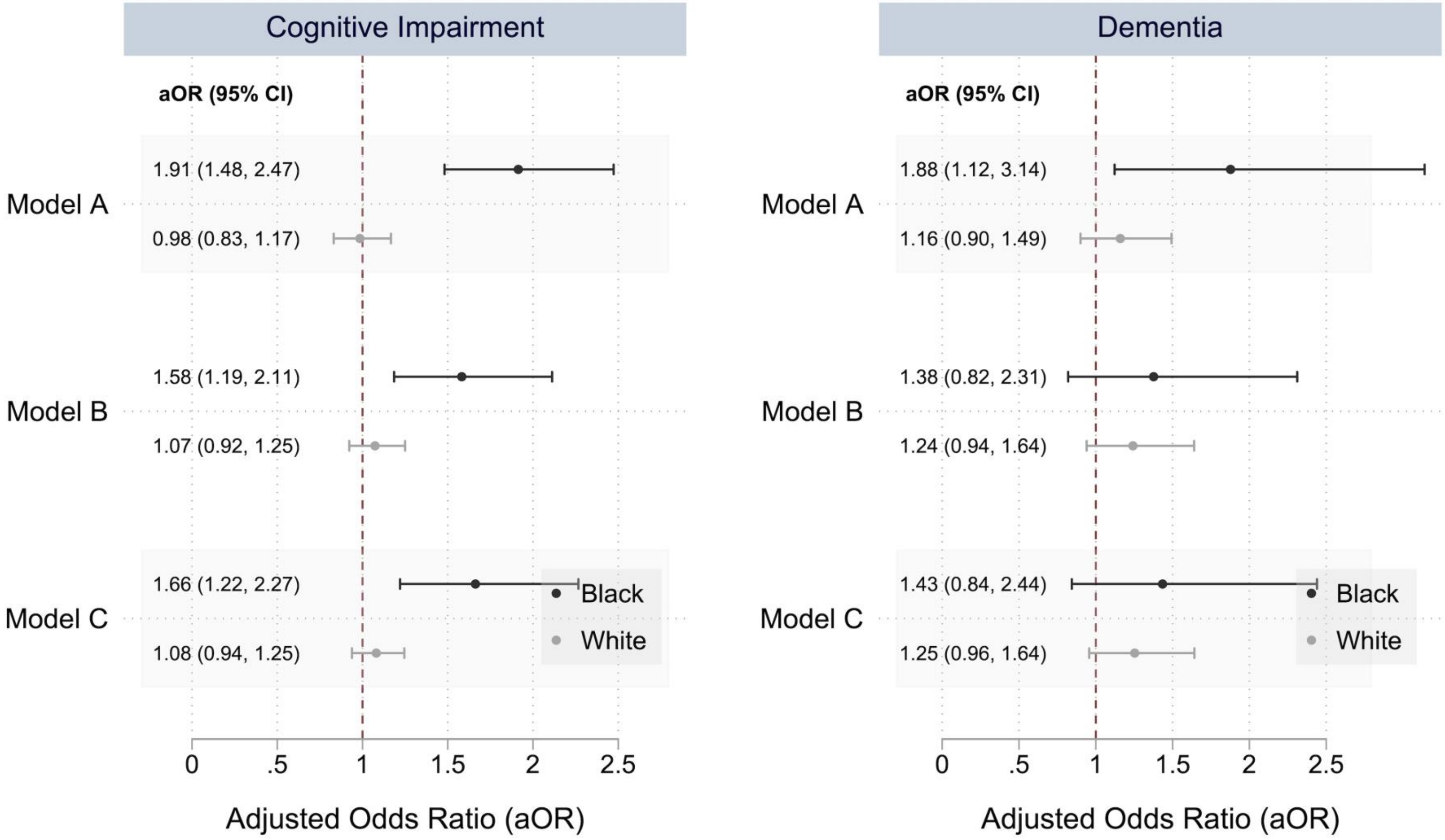

Notes: Multilevel regressions were used to estimate the association between school segregation and cognitive impairment (left panel) and dementia (right panel), for Black (in black color) and White participants (in gray color) in the Health and Retirement Study (HRS, 1995-2018). Black refers to non-Hispanic Black, and White refers to non-Hispanic White. Horizontal lines represent the 95% confidence

© 2025 Lin Z et al. *JAMA Network Open.*

interval, and numerical estimates are displayed alongside each line. In the sensitivity analyses, we restricted our sample to participants who lived in urban areas during childhood. The segregation measure is the same as the main setting. Model A adjusted for covariates, including age, sex, parental education, childhood residence in U.S. Southern States, regional indicators for childhood residence, a birth-year trend indicator, and region-specific birth-year trend interactions. Model B additionally included early-life mediator, i.e., educational attainment. Model C further added mid-life mediators, including health factors involving hypertension, diabetes, heart diseases, psychiatric conditions, obesity, and smoking behaviors. Random intercepts were included at the state level to account for unobserved heterogeneity and differences between states, while individual-level random intercepts addressed within-individual correlations across multiple observations. Robust standard errors, clustered at the state level, were estimated accounting for within-state correlation.

© 2025 Lin Z et al. *JAMA Network Open.*

eFigure 11. Sensitivity analysis using self-reported time-varying measures of school segregation exposure: association between school segregation and cognitive score by race

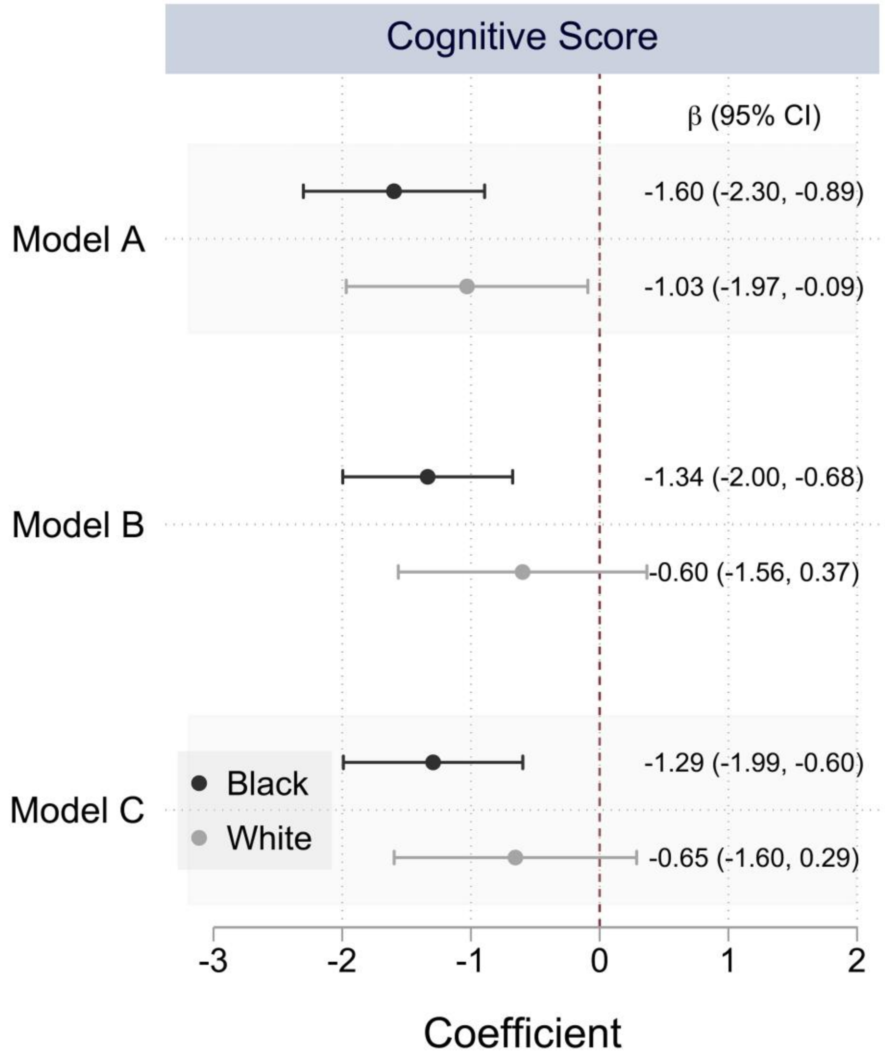

© 2025 Lin Z et al. *JAMA Network Open.*

Notes: Multilevel regression models were used to estimate the association between school segregation and cognitive score for Black (in black color) and White participants (in gray color) in the Health and Retirement Study (HRS, 1995-2018). Black refers to non-Hispanic Black, and White refers to non-Hispanic White. Horizontal lines represent the 95% confidence interval, and numerical estimates are displayed alongside each line. In the sensitivity analyses, we used a more time-varying, self-reported measure of school segregation from the HRS life history survey. Specifically, HRS participants who completed the life history mailed survey in 2015-2017 were asked to report the schools they attended during their primary education and whether the majority of children in each school were White, Black, Hispanic or others. School was classified as segregated if most children in the school were Black, Hispanic or others. If the schools the participants attended during primary education were segregated schools, they were coded as 1 (and 0 otherwise). Model A adjusted for covariates, including age, sex, parental education, childhood residence in U.S. Southern States, regional indicators for childhood residence, a birth-year trend indicator, and region-specific birth-year trend interactions. Model B additionally included early-life mediator, i.e., educational attainment. Model C further added mid-life mediators, including health factors involving hypertension, diabetes, heart diseases, psychiatric conditions, obesity, and smoking behaviors. Random intercepts were included at the state level to account for unobserved heterogeneity and differences between states, while individual-level random intercepts addressed within-individual correlations across multiple observations. Robust standard errors, clustered at the state level, were estimated accounting for within-state correlation.

© 2025 Lin Z et al. *JAMA Network Open.*

eFigure 12. Sensitivity analysis using self-reported time-varying measures of school segregation exposure: association between school segregation and cognitive impairment and dementia by race

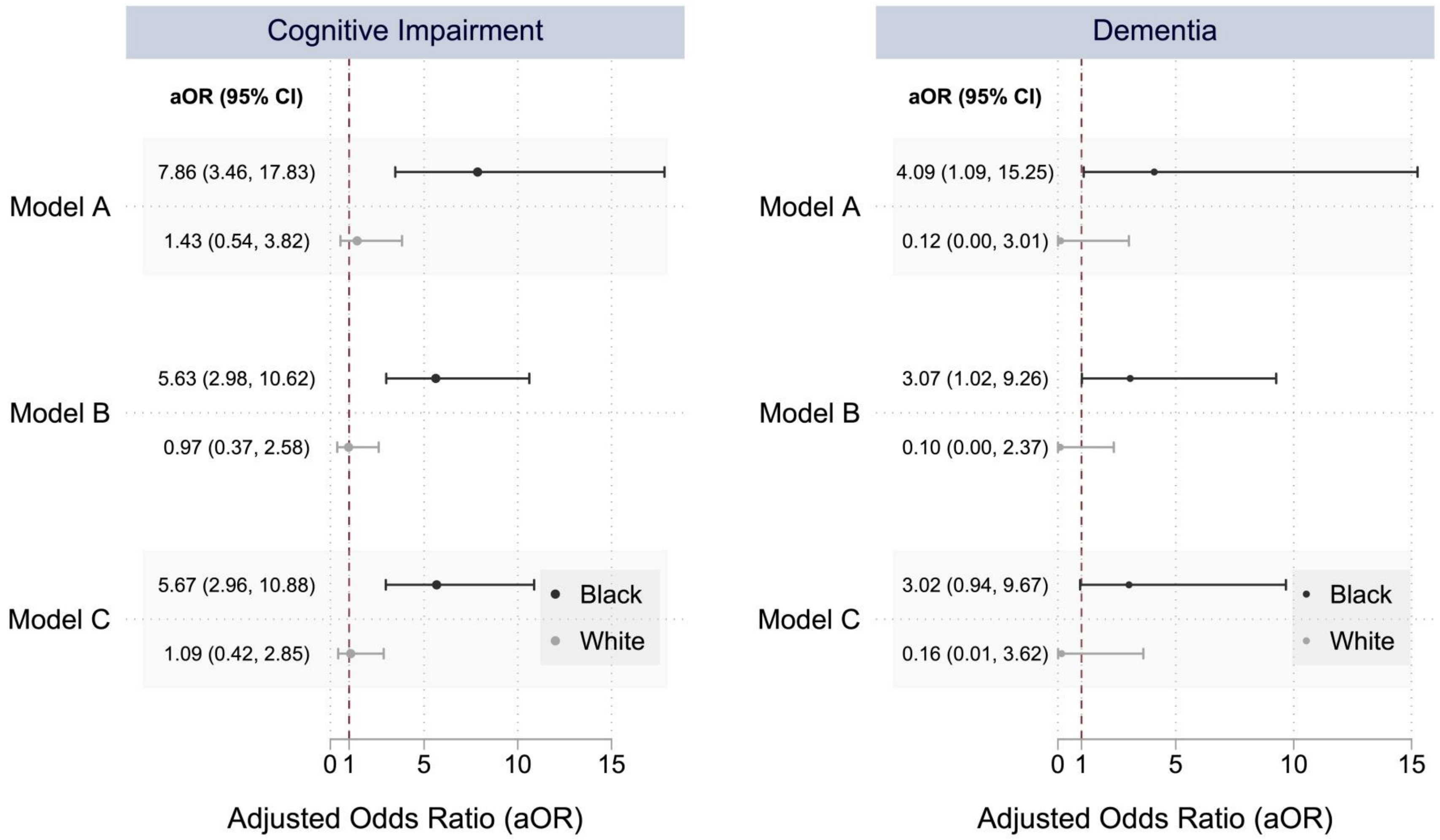

Notes: Multilevel regressions were used to estimate the association between school segregation and cognitive impairment (left panel) and dementia (right panel), for Black (in black color) and White participants (in gray color) in the Health and Retirement Study (HRS, 1995-2018). Black refers to non-Hispanic Black, and White refers to non-Hispanic White. Horizontal lines represent the 95% confidence

© 2025 Lin Z et al. *JAMA Network Open.*

interval, and numerical estimates are displayed alongside each line. In the sensitivity analyses, we used a more time-varying, self-reported measure of school segregation from the HRS life history survey. Specifically, HRS participants who completed the life history mailed survey in 2015-2017 were asked to report the schools they attended during their primary education and whether the majority of children in each school were White, Black, Hispanic or others. School was classified as segregated if most children in the school were Black, Hispanic or others. If the schools the participants attended during primary education were segregated schools, they were coded as 1 (and 0 otherwise). Model A adjusted for covariates, including age, sex, parental education, childhood residence in U.S. Southern States, regional indicators for childhood residence, a birth-year trend indicator, and region-specific birth-year trend interactions. Model B additionally included early-life mediator, i.e., educational attainment. Model C further added mid-life mediators, including health factors involving hypertension, diabetes, heart diseases, psychiatric conditions, obesity, and smoking behaviors. Random intercepts were included at the state level to account for unobserved heterogeneity and differences between states, while individual-level random intercepts addressed within-individual correlations across multiple observations. Robust standard errors, clustered at the state level, were estimated accounting for within-state correlation.

© 2025 Lin Z et al. *JAMA Network Open.*

eFigure 13. Sensitivity analysis using self-reported time-varying measures of school segregation exposure with additional adjustment for state-level geographical and temporal variations: association between school segregation and cognitive score by race

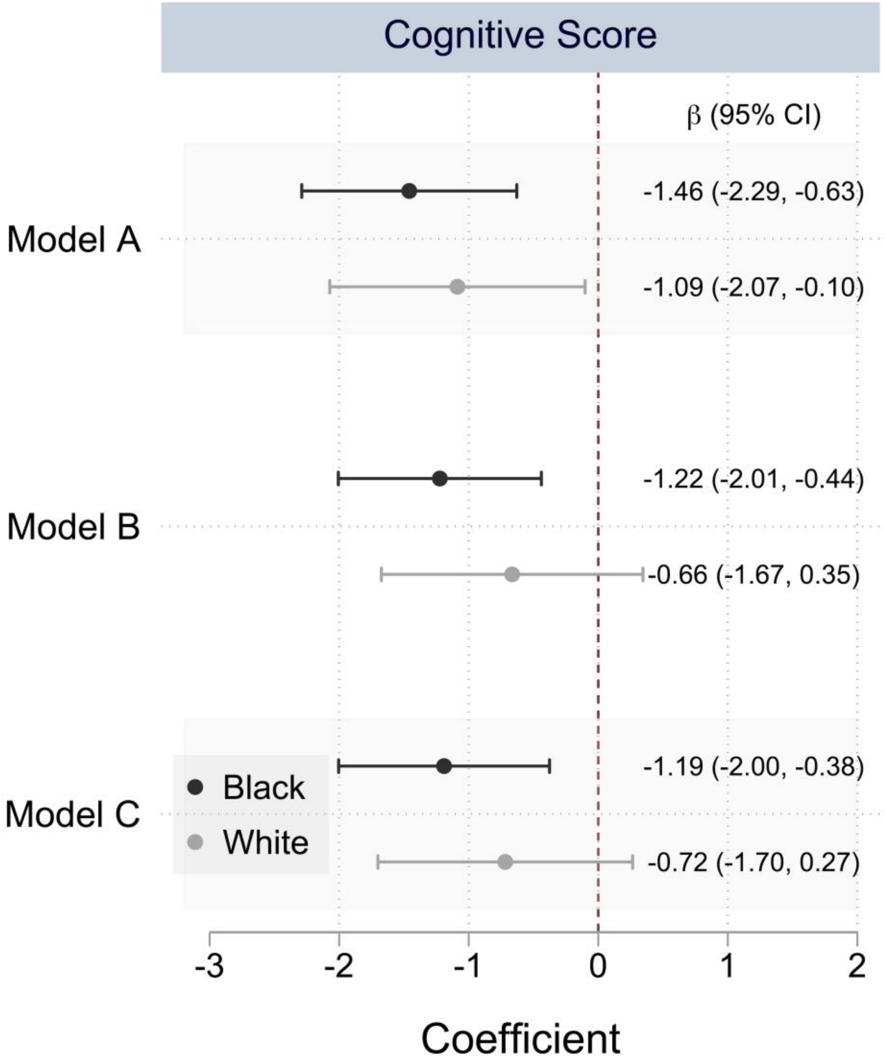

© 2025 Lin Z et al. *JAMA Network Open.*

Notes: Multilevel regression models were used to estimate the association between school segregation and cognitive score for Black (in black color) and White participants (in gray color) in the Health and Retirement Study (HRS, 1995-2018). Black refers to non-Hispanic Black, and White refers to non-Hispanic White. Horizontal lines represent the 95% confidence interval, and numerical estimates are displayed alongside each line. In the sensitivity analyses, we used a more time-varying, self-reported measure of school segregation from the HRS life history survey (If the schools the participants attended during primary education were segregated schools, they were coded as 1 and 0 otherwise). Moreover, we introduced state-level birth-year trend indicators to account for potential geographical and time-varying confounding. Model A adjusted for covariates, including age, sex, parental education, childhood residence in U.S. Southern States, regional indicators for childhood residence, a birth-year trend indicator, and region-specific birth-year trend interactions. Model B additionally included early-life mediator, i.e., educational attainment. Model C further added mid-life mediators, including health factors involving hypertension, diabetes, heart diseases, psychiatric conditions, obesity, and smoking behaviors. Random intercepts were included at the state level to account for unobserved heterogeneity and differences between states, while individual-level random intercepts addressed within-individual correlations across multiple observations. Robust standard errors, clustered at the state level, were estimated accounting for within-state correlation.

© 2025 Lin Z et al. *JAMA Network Open.*

eFigure 14. Sensitivity analysis using self-reported time-varying measures of school segregation exposure with additional adjustment for state-level geographical and temporal variations: association between school segregation and cognitive impairment and dementia by race

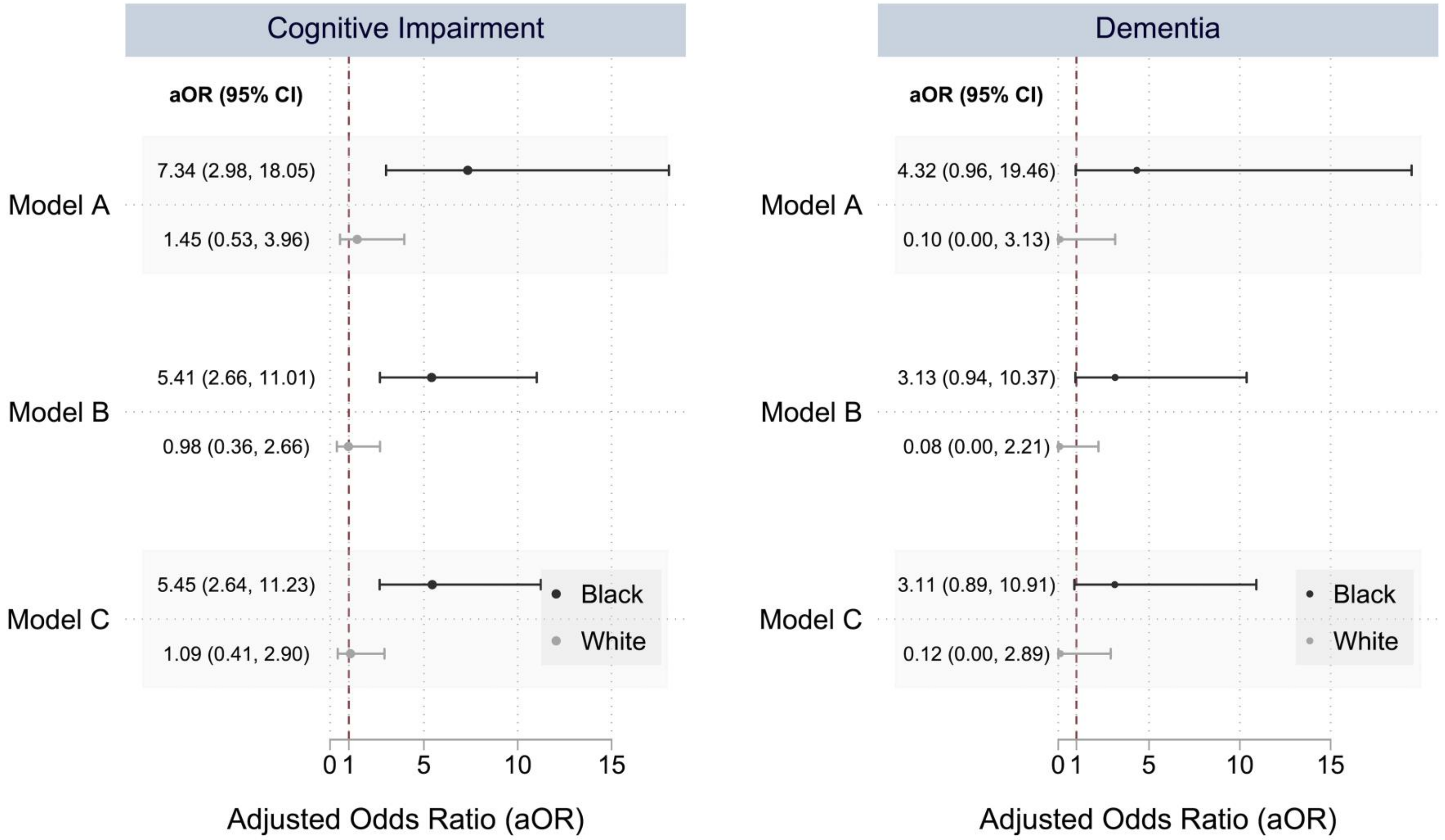

Notes: Multilevel regressions were used to estimate the association between school segregation and cognitive impairment (left panel) and dementia (right panel), for Black (in black color) and White participants (in gray color) in the Health and Retirement Study (HRS,

© 2025 Lin Z et al. *JAMA Network Open.*

1995-2018). Black refers to non-Hispanic Black, and White refers to non-Hispanic White. Horizontal lines represent the 95% confidence interval, and numerical estimates are displayed alongside each line. In the sensitivity analyses, we used a more time-varying, self-reported measure of school segregation from the HRS life history survey (If the schools the participants attended during primary education were segregated schools, they were coded as 1 and 0 otherwise). Moreover, we introduced state-level birth-year trend indicators to account for potential geographical and time-varying confounding. Model A adjusted for covariates, including age, sex, parental education, childhood residence in U.S. Southern States, regional indicators for childhood residence, a birth-year trend indicator, and region-specific birth-year trend interactions. Model B additionally included early-life mediator, i.e., educational attainment. Model C further added mid-life mediators, including health factors involving hypertension, diabetes, heart diseases, psychiatric conditions, obesity, and smoking behaviors. Random intercepts were included at the state level to account for unobserved heterogeneity and differences between states, while individual-level random intercepts addressed within-individual correlations across multiple observations. Robust standard errors, clustered at the state level, were estimated accounting for within-state correlation.

© 2025 Lin Z et al. *JAMA Network Open.*

eTable 1. Differences in characteristics between sample with high vs. low level of school segregation assessed using appropriate statistical tests

	Low Segregation (N=80,127)	High Segregation (N=26,851)	*P*-values [a]
**School Segregation**			
Dissimilarity Index (0-100), mean (SD)	77.5 (4.6)	86.9 (1.9)	<0.001
**Cognitive Outcomes**			
Cognitive Score (0-27), mean (SD)	14.5 (4.5)	13.6 (4.9)	<0.001
Cognitive Impairment, No. (%)	22432 (28.0)	9924 (37.0)	<0.001
Dementia, No. (%)	7448 (9.3)	3790 (14.1)	<0.001
**Covariates**			
Age, mean (SD), y	75.7 (7.5)	75.6 (7.7)	0.13
Female, No. (%)	46341 (57.8)	15846 (59.0)	<0.001
Race			<0.001
Non-Hispanic Black, No. (%)	9003 (11.2)	7101 (26.4)	
Non-Hispanic White, No. (%)	71124 (88.8)	19750 (73.6)	
Mother's Education			<0.001
<8 years, No. (%)	16358 (20.4)	7180 (26.7)	
8-12 years, No. (%)	48177 (60.1)	14732 (54.9)	
>12 years, No. (%)	7635 (9.5)	2005 (7.5)	
Unknown, No. (%)	7957 (9.9)	2934 (10.9)	
Father's Education			<0.001
<8 years, No. (%)	20373 (25.4)	8467 (31.5)	
8-12 years, No. (%)	41313 (51.6)	12330 (45.9)	
>12 years, No. (%)	7667 (9.6)	1882 (7.0)	

© 2025 Lin Z et al. *JAMA Network Open.*

Unknown, No. (%)	10774 (13.4)	4172 (15.5)	
Childhood Residence in Southern States, No. (%)	22446 (28.0)	15894 (59.2)	<0.001
**Early-Life Mediators - Education**			
Years of Educational Attainment, mean (SD)	12.6 (2.8)	11.8 (3.4)	<0.001
**Mid-Life Mediators - Health Factors**			
Hypertension, No. (%)	48732 (60.8)	17079 (63.6)	<0.001
Diabetes, No. (%)	16469 (20.6)	5863 (21.8)	<0.001
Heart Diseases, No. (%)	25701 (32.1)	8739 (32.5)	0.15
Psychiatric Conditions, No. (%)	11450 (14.3)	3997 (14.9)	0.016
Obesity, No. (%)	26917 (33.6)	9652 (35.9)	<0.001
Smoking			<0.001
Never smoking, No. (%)	33616 (42.0)	12380 (46.1)	
Ever smoking, No. (%)	39341 (49.1)	11873 (44.2)	
Currently smoking, No. (%)	7170 (8.9)	2598 (9.7)	

Abbreviations: SD=standard deviation, ADL=activities of daily living, IADL=instrumental activities of daily living.

[a] Differences in characteristics between sample with high and low level of school segregation were assessed using appropriate statistical tests: Chi-square tests for categorical variables and Welch t-tests for continuous variables.

© 2025 Lin Z et al. *JAMA Network Open.*

eTable 2. Association between school segregation and cognitive outcomes for Black participants in the HRS (1995-2018)

	Black Participants			
VARIABLES	Model A	Model B	Model C	Association mediated by early-life and mid-life factors
**Panel A. Cognitive Score (0-27)**	β Coefficient (95% CI)			
School Segregation	-0.95<br>(-1.24, -0.67)	-0.24<br>(-0.40, -0.07)	-0.26<br>(-0.43, -0.09)	73%
Observations	14,209	14,209	14,209	
**Panel B. Cognitive Impairment (0/1)**	Adjusted Odds Ratio (95% CI)			
School Segregation	2.03<br>(1.64, 2.51)	1.31<br>(1.11, 1.54)	1.35<br>(1.12, 1.63)	58%
Observations	16,104	16,104	16,104	
**Panel C. Dementia (0/1)**	Adjusted Odds Ratio (95% CI)			
School Segregation	2.04<br>(1.57, 2.66)	1.26<br>(1.02, 1.56)	1.26<br>(1.03, 1.54)	67%
Observations	16,104	16,104	16,104	
Covariates	YES	YES	YES	
Mediators: Educational Attainment	NO	YES	YES	
Mediators: Health Factors	NO	NO	YES	

Notes: Multilevel regressions were used to estimate the association associations between school segregation and cognitive outcomes, including cognitive score (0-27) (Panel A), cognitive impairment (0/1) (Panel B), and dementia (0/1) (Panel C) for Black participants in

© 2025 Lin Z et al. *JAMA Network Open.*

the Health and Retirement Study (HRS, 1995-2018). Black refers to non-Hispanic Black, and White refers to non-Hispanic White. Model A adjusted for covariates, including age, sex, parental education, childhood residence in U.S. Southern States, regional indicators for childhood residence, a birth-year trend indicator, and region-specific birth-year trend interactions. Model B additionally included early-life mediator, i.e., educational attainment. Model C further added mid-life mediators, including health factors involving hypertension, diabetes, heart diseases, psychiatric conditions, obesity, and smoking behaviors. Random intercepts were included at the state level to account for unobserved heterogeneity and differences between states, while individual-level random intercepts addressed within-individual correlations across multiple observations. Robust standard errors, clustered at the state level, were estimated accounting for within-state correlation. Mediation was evaluated using the difference method (percentage reduction), which compares the coefficients from the mediated model (i.e., Model C) to the unmediated model (i.e., Model A). The percentage reduction of the coefficients reflects the extent to which mediators explain the association between school segregation and cognitive outcomes, and the results are listed at the right column.

© 2025 Lin Z et al. *JAMA Network Open.*

eTable 3. Association between school segregation and cognitive outcomes for White participants in the HRS (1995-2018)

	White Participants			
VARIABLES	Model A	Model B	Model C	Association mediated by early-life and mid-life factors
**Panel A. Cognitive Score (0-27)**		β Coefficient (95% CI)		
School Segregation	-0.15<br>(-0.40, 0.10)	-0.14<br>(-0.30, 0.02)	-0.13<br>(-0.28, 0.01)	10%
Observations	83,205	83,205	83,205	
**Panel B. Cognitive Impairment (0/1)**		Adjusted Odds Ratio (95% CI)		
School Segregation	1.14<br>(0.93, 1.40)	1.16<br>(1.03, 1.32)	1.15<br>(1.03, 1.29)	-9%
Observations	90,874	90,874	90,874	
**Panel C. Dementia (0/1)**		Adjusted Odds Ratio (95% CI)		
School Segregation	1.13<br>(0.91, 1.40)	1.13<br>(0.92, 1.38)	1.11<br>(0.93, 1.33)	14%
Observations	90,874	90,874	90,874	
Covariates	YES	YES	YES	
Mediators: Educational Attainment	NO	YES	YES	
Mediators: Health Factors	NO	NO	YES	

Notes: Multilevel regressions were used to estimate the association associations between school segregation and cognitive outcomes, including cognitive score (0-27) (Panel A), cognitive impairment (0/1) (Panel B), and dementia (0/1) (Panel C) for White participants in

© 2025 Lin Z et al. *JAMA Network Open.*

the Health and Retirement Study (HRS, 1995-2018). Black refers to non-Hispanic Black, and White refers to non-Hispanic White. Model A adjusted for covariates, including age, sex, parental education, childhood residence in U.S. Southern States, regional indicators for childhood residence, a birth-year trend indicator, and region-specific birth-year trend interactions. Model B additionally included early-life mediator, i.e., educational attainment. Model C further added mid-life mediators, including health factors involving hypertension, diabetes, heart diseases, psychiatric conditions, obesity, and smoking behaviors. Random intercepts were included at the state level to account for unobserved heterogeneity and differences between states, while individual-level random intercepts addressed within-individual correlations across multiple observations. Robust standard errors, clustered at the state level, were estimated accounting for within-state correlation. Mediation was evaluated using the difference method (percentage reduction), which compares the coefficients from the mediated model (i.e., Model C) to the unmediated model (i.e., Model A). The percentage reduction of the coefficients reflects the extent to which mediators explain the association between school segregation and cognitive outcomes, and the results are listed at the right column.

© 2025 Lin Z et al. *JAMA Network Open.*

eAppendix. Sensitivity Analyses

A comprehensive series of sensitivity analyses were conducted to ensure the robustness of our findings and to assess the associations between exposure to school segregation and cognitive outcomes across varying levels and specifications of exposure.

1. Less Extreme Cutoff for High Segregation

First, we redefined the threshold for high levels of school segregation, using a less extreme cutoff (the top tertile instead of the top quintile). This adjustment allowed us to examine whether the associations between segregation and cognitive outcomes persisted when considering a broader range of segregation levels. The main patterns held as shown in eFigures 5-6, though the associations were relatively smaller compared to our main specification (top quintile). Combined with the patterns shown in eFigures 3-4, these results suggest that individuals exposed to the most extreme levels of segregation (as captured by the top quintile) tend to experience disproportionately worse cognitive outcomes.

2. Continuous Measure of School Segregation

Second, we employed a continuous measure of school segregation, represented by the dissimilarity index, rather than using a dichotomous classification (low vs. high segregation). While this approach assumes a linear relationship, it captures more granular variations in segregation. As shown in eFigures 7-8, the results supported our finding that higher segregation levels are associated with poorer cognitive outcomes. The relatively smaller associations observed using the continuous specification further imply that the strongest effects of school

segregation may occur among individuals exposed to extreme segregation, as reflected by the top quintile.

3. Restriction to Urban Sample

Third, recognizing that the dissimilarity index is based on metropolitan data, we restricted our sample to participants who lived in urban areas during childhood. This restriction allowed for a more targeted assessment of segregation. Our results remained consistent with the main findings (eFigures 9-10), further confirming the robustness of our analysis.

4. Time-Varying Measure of Segregation

Fourth, we incorporated a more time-varying, self-reported measure of school segregation from the HRS life history survey. Specifically, HRS participants who completed the life history mailed survey in 2015-2017 were asked to report the schools they attended during their primary education and whether the majority of children in each school were White, Black, Hispanic or others. School was classified as segregated if most children in the school were Black, Hispanic or others. If the schools the participants attended during primary education were segregated schools, they were coded as 1 (and 0 otherwise). This self-reported measure, validated in previous research, provided a time-varying evaluation of participants' exposure to segregation. Although it cannot capture the relative intensity of the exposure, it offered an additional layer of temporal variation. Results from the analysis (eFigures 11-12) aligns with our primary findings, further reinforcing the association between school segregation and cognitive outcomes.

5. Additional Adjustment for State-level Geographical and Temporal Variations

© 2025 Lin Z et al. *JAMA Network Open.*

Finally, we introduced state-level birth-year trend indicators to account for potential geographical and time-varying confounding. Using the self-reported life history measure of school segregation, which incorporates temporal variation, enabled us to add state-level indicators to adjust for differences across various time periods and regions. The consistent findings shown in eFigures 13-14 further strengthened our main results.

These sensitivity analyses collectively confirmed the robustness of our findings. Moreover, although the missing data in our study sample is minimal (<1%), we performed additional robustness checks using multiple imputation, and the estimates remained nearly identical, providing further confidence in the consistency of our results.

© 2025 Lin Z et al. *JAMA Network Open.*
