# Supplementary material for: Exposure to School Racial Segregation and Late-Life Cognitive Outcomes
Source: JAMA Netw Open. 2025 Jan 3;8(1):e2452713. doi: 10.1001/jamanetworkopen.2024.52713 (PMC11699536; doi:10.1001/jamanetworkopen.2024.52713)
Supplement: Supplement 2. — Data Sharing Statement [file jamanetwopen-e2452713-s002.pdf]

Data Sharing Statement

Lin. Exposure to School Racial Segregation and Late-Life Cognitive Outcomes. *JAMA Netw Open*. Published December 30, 2024. doi:10.1001/jamanetworkopen.2024.52713

Data
Data available: Yes
Data types: Deidentified participant data
How to access data: https://hrs.isr.umich.edu/
When available: With publication

Supporting Documents
Document types: Statistical/analytic code
How to access documents: xi.chen@yale.edu
When available: With publication

Additional Information
Who can access the data: researchers whose proposed use of the data has been approved
Types of analyses: for a specified purpose
Mechanisms of data availability: after approval of a proposal
Any additional restrictions: no
